# Supplementary material for: txci-ATAC-seq: a massive-scale single-cell technique to profile chromatin accessibility
Source: Genome Biol. 2024 Mar 22;25:78. doi: 10.1186/s13059-023-03150-1 (PMC10958877; doi:10.1186/s13059-023-03150-1)
Supplement: Supplementary file 1 — Additional file 1: Fig S1. Collision rates of standard 10X protocol coupled with combinatorial indexing. Fig S2. Exponential amplification during GEM PCR enables deconvolution of cells in the same droplet. Fig S3. Evaluating the performance of txci-ATAC-seq on brain samples. Fig S4. Consistency of scATAC-seq mouse brain datasets. Fig S5. Quality metrics of txci-ATAC-seq in the native lung and liver samples with loading 100,000 or 200,000 nuclei. Fig S6. Cell type annotation of mouse lung samples. Fig S7. Cell type annotation of mouse liver samples. Fig S8. Cell type annotation of human lung sample with label transfer. Fig S9. Comparison of prediction accuracy between txci-ATAC-seq and sci-ATAC-seq in mouse lung cells. Fig S10. Characterization of cellular heterogeneity in human lung tissue. Fig S11. txci-ATAC-seq are robust to batch effects. Fig S12. Comparison of txci-ATAC with dsciATAC using a bead-merging strategy. Fig S13. Phased-txci-ATAC-seq improves multiplexing capability without sacrificing data quality. Fig S14. Chromatin accessibility changes induced by CC16-/- deficiency in mouse lung. Fig S15. Functional analysis and regulatory variant identification in CC16 deficient mouse. Fig S16. SNP-driven differences in motif usage alter chromatin accessibility. Fig S17. Comparison of empirical and theoretical cell recovery between molecular and cellular hashing strategies. Fig S18. Examination of efficiency for in-droplet and sample index PCR. [file 13059_2023_3150_MOESM1_ESM.docx]

**Additional file 1: Fig S1-S18.**

**
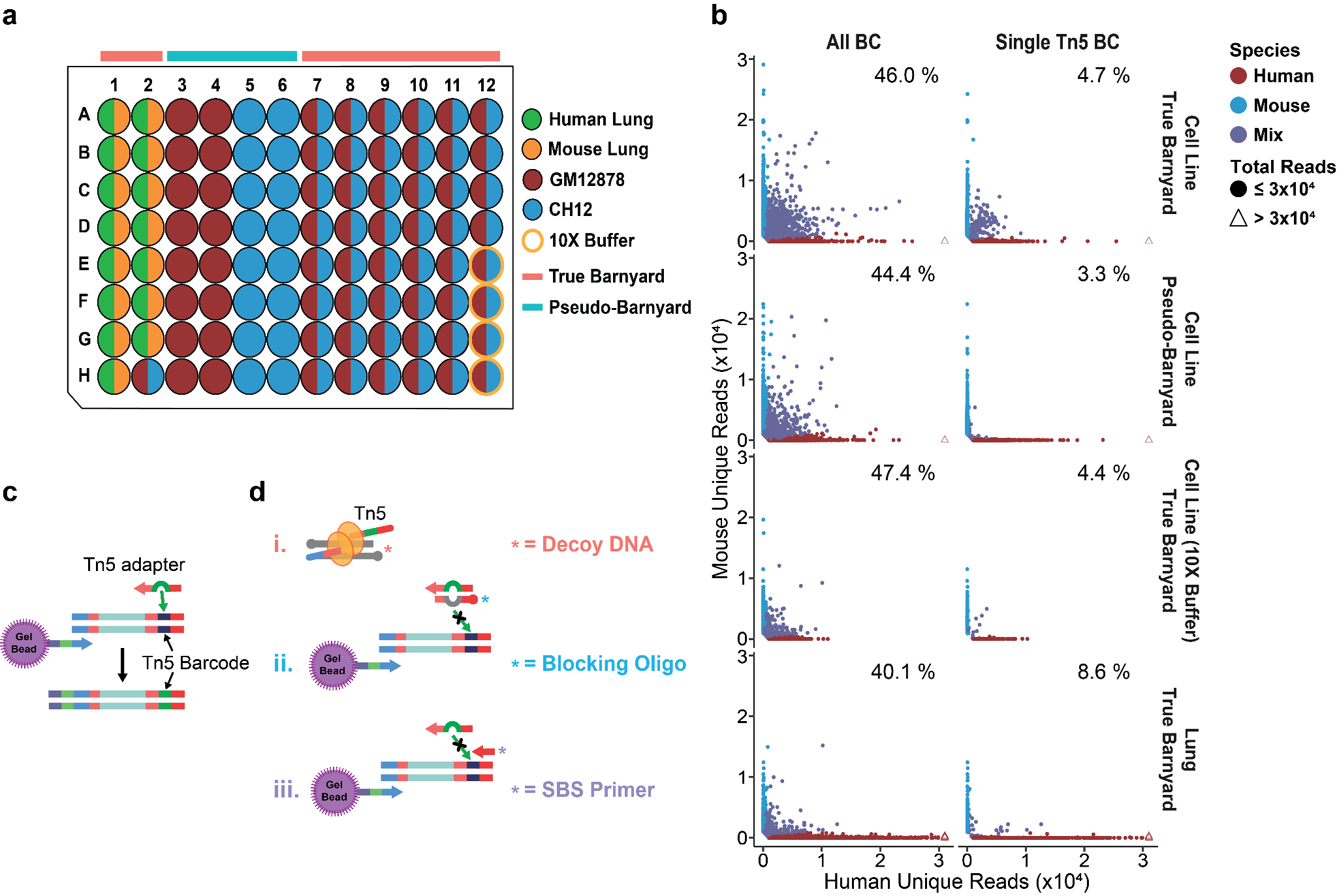
**

**Fig S1. Collision rates of standard 10X protocol coupled with combinatorial indexing.** a) Well assignment for each cell source and barnyard design. The wells with a mixture of species are shown as half-circles of two different colors corresponding to each species. The wells where tagmentation was done with the 10X ATAC buffer are highlighted by orange outer circles. b) The scatter plots showing the number of reads mapped to either the human or mouse genome for each barnyard design. The plots on the left-hand side include all cell barcodes, and the plots on the right-hand side only visualize the 10X bead barcodes associated with a single Tn5 barcode. The percentage represents the estimated collision rate for each plot. The x- and y-axes are capped at 3x10^4^ reads, and the cells with more than 3x10^4^ reads are denoted by triangles. c) The theoretical model of the process of Tn5 barcode swapping. During in-droplet PCR, the barcodes present within the remaining unincorporated Tn5 adapters replace the original Tn5 barcodes tagging the DNA fragment from the tagmentation step. d) The proposed strategies to block barcode swapping.


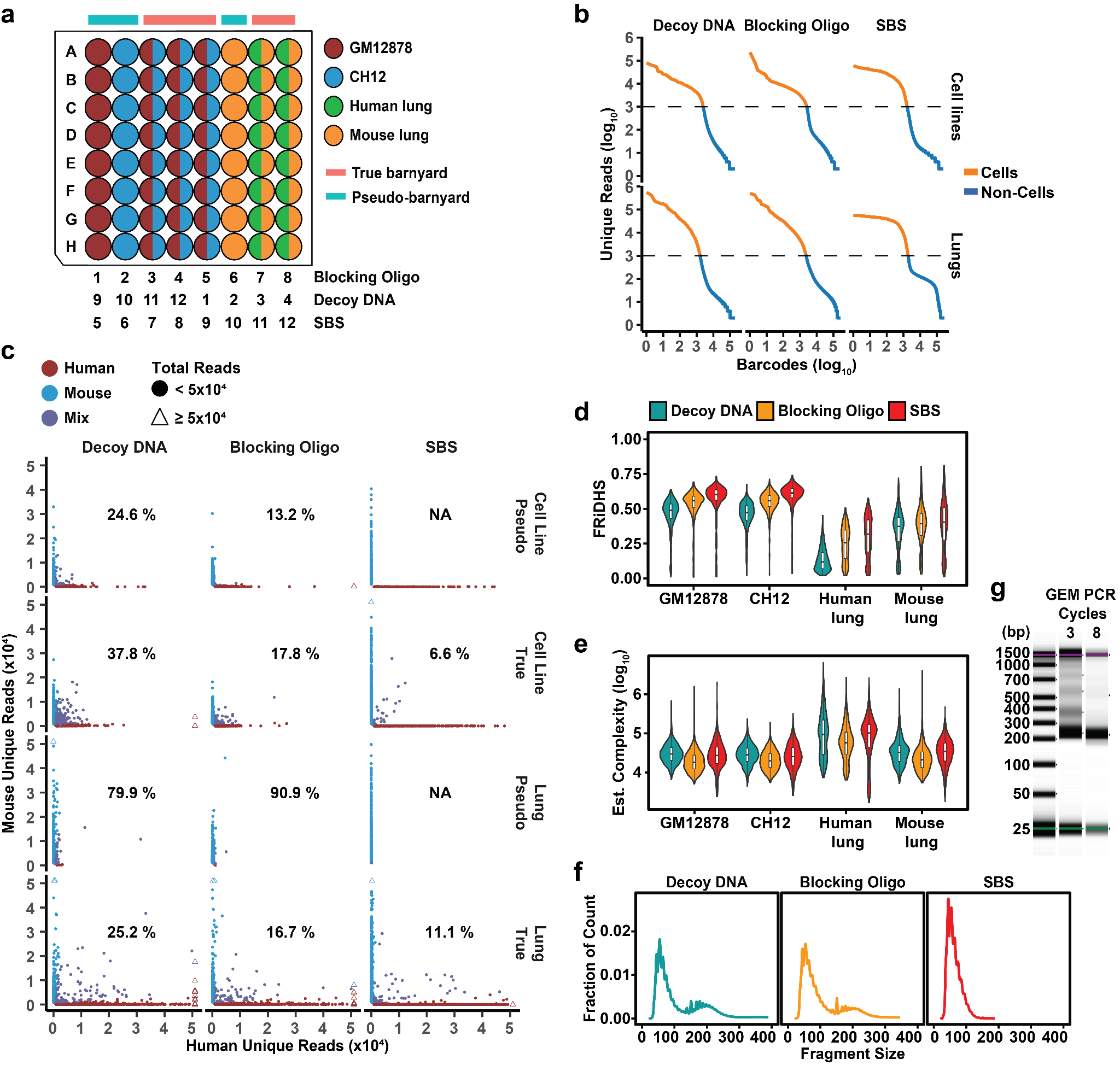


**Fig S2. Exponential amplification during GEM PCR enables deconvolution of cells in the same droplet.** a) Well assignments for the experiment testing the performance of each blocking strategy. Each blocking condition was allocated to ⅔ of the plate and a total of two 96-well plates were used to test all three conditions. The column ID assigned to each blocking condition is shown under the 96-well plate. b) “Knee” plots showing the separation between cell barcodes (orange line) and background barcodes (blue line) in either cell lines or lung tissues using different blocking methods. The dashed line indicates the threshold (1000 reads) used to identify cell barcodes. c) Scatter plots showing the number of reads mapped to either human or mouse genome for the true barnyard (True) and pseudo-barnyard (Pseudo) settings with a mixture of GM12878 and CH12 cell lines or human and mouse lung cells at each blocking condition. Only mouse lung cells were loaded to the wells assigned to the “lung pseudo-barnyard”. The percentage represents the estimated collision rate (including doublets originating from the same species). The x- and y-axes are capped at 5x10^4^ reads, and the cells with at least 5x10^4^ reads are denoted by triangles. d-e) QC metrics of each blocking approach across different cell sources. The (d) FRiDHS and (e) estimated complexity (on a log_10_ scale) in each cell barcode are plotted for each strategy. f) The fragment size distribution of each blocking condition. g) TapeStation gel image showing the fragment size distributions at either 3 or 8 GEM PCR cycles. The first lane on the left indicates the DNA ladder.

**
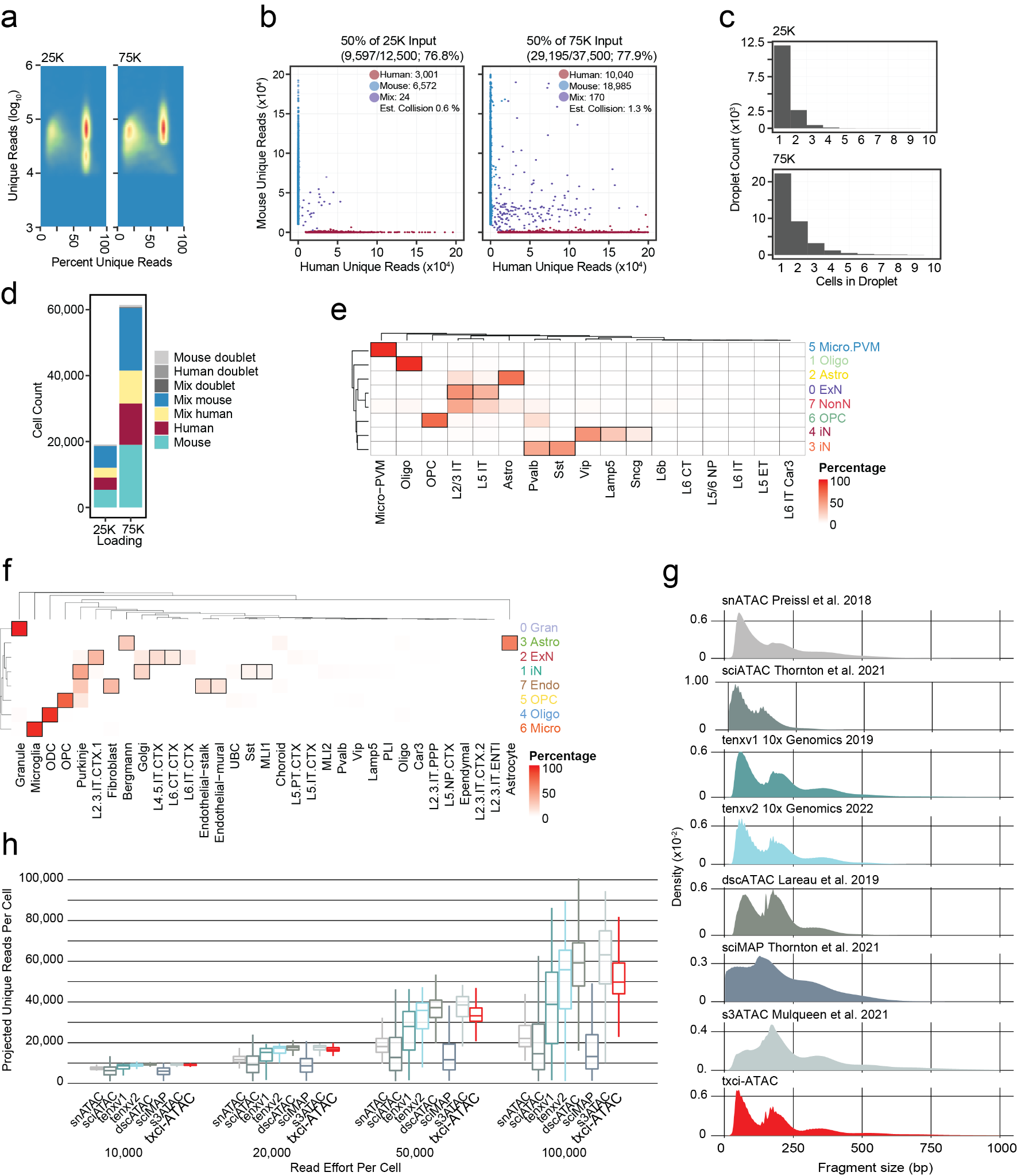
**

**Fig S3. Evaluating the performance of txci-ATAC-seq on brain samples.** a) Two-dimensional density map of cells passing initial read filters for percent unique reads (library saturation) and unique read counts. b) Mixed-species tagmentation wells were subject to alignment in both human and mouse reference genomes. c) Number of cells per droplet quantified on a histogram, showing a majority of droplets still contain only a single cell. d) Quantification of cells in 25,000 and 75,000 library pools. Conditions include doublets uncovered either through cross-species alignment (“mixed doublet”) or through a reduced dimension detection strategy (see Methods). Other cells passing these filters are colored by identified species and tagmentation conditions. e,f) Hierarchically clustered heatmap of cell identification in the human cortex (e) and mouse whole brain (f) samples using gene activity scores for label transfer. The Brain Map M1 Cortex RNA dataset was used to annotate human cells and the Brain Map mouse cortex and hippocampus RNA dataset and mouse cerebellum (GSE165371) were used to annotate mouse cells. Values reflect the percentage of cells per cluster with each label as its maximum predicted value. g) Density plots of fragment length for fragments ranging from 1-1000 bp per technology. h) Grouped boxplots of projected unique read count per sequencing effort for each cell by technology.


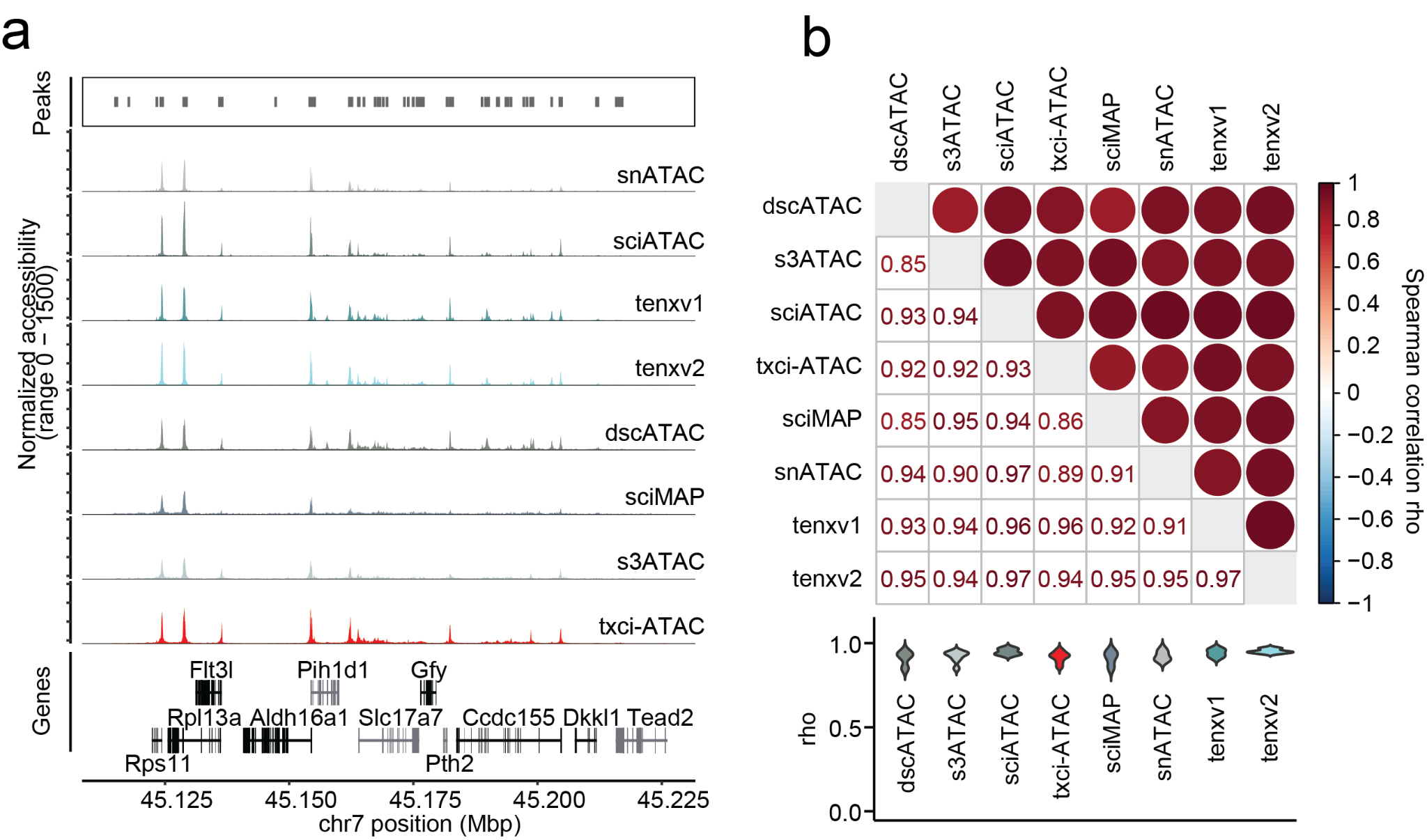


**Fig S4. Consistency of scATAC-seq mouse brain datasets.** a) Aggregated chromatin accessibility at the *Slc17a7* gene body and extended region (50,000 bp upstream and downstream) across methods. The aggregated accessibility signal for each cluster was normalized by a scaling factor computed as the number of cells in the cluster multiplied by the mean sequencing depth for the cells in that technology. Peaks called on all technologies aggregated are shown above and gene locations are shown below. b) Spearman correlation rho values shown across technologies in a pairwise fashion. Average gene accessibility (see Methods) was correlated and plotted both as a colored dot plot (top triangle) and as the rho value (bottom triangle). The distribution of the correlation coefficient for each technique was visualized using a violin plot.

**
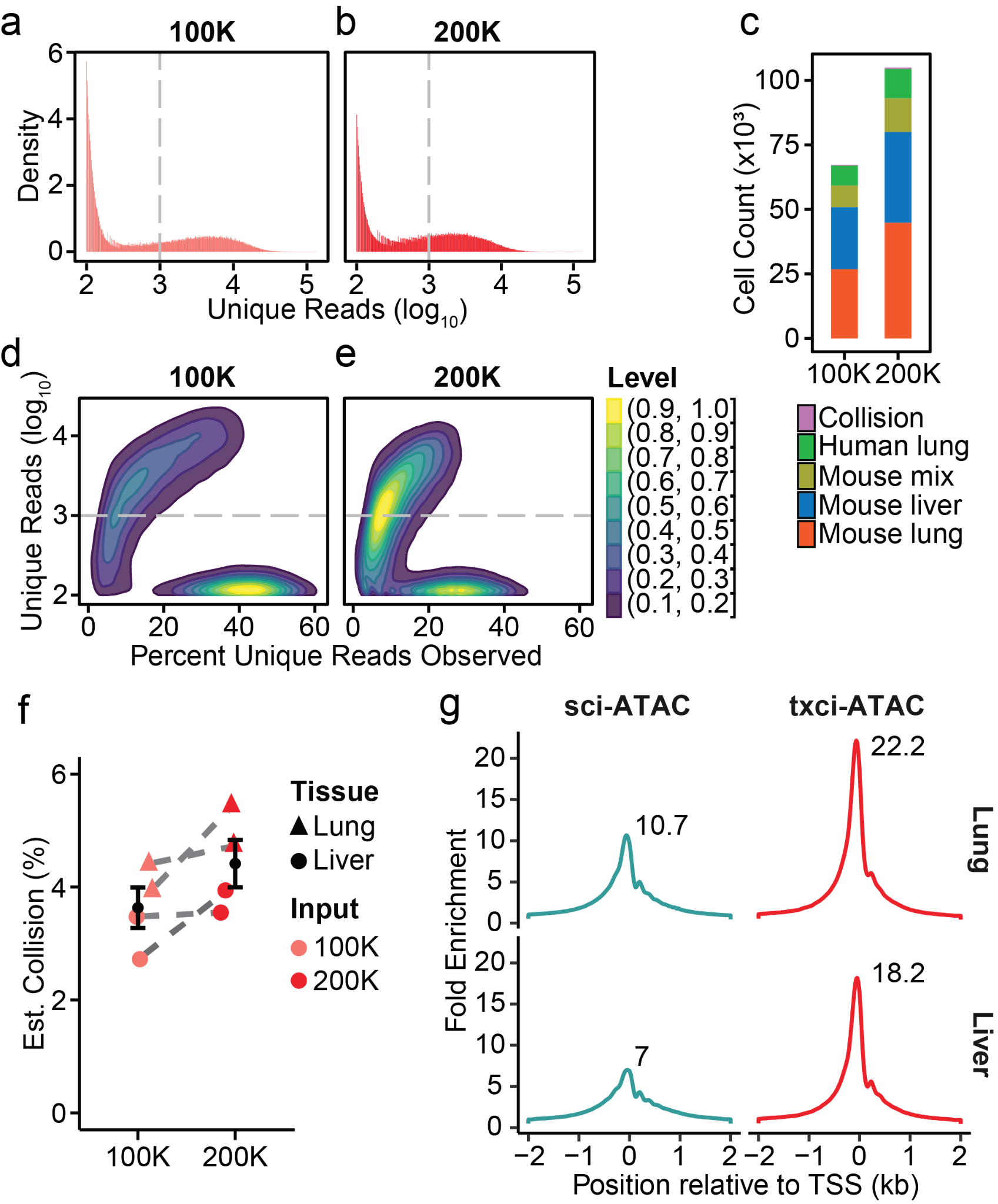
**

**Fig S5. Quality metrics of txci-ATAC-seq in the native lung and liver samples with loading 100,000 or 200,000 nuclei.** a,b) Histograms showing the distribution of unique read counts (on a log_10_ scale) assigned to each possible barcode combination at the 100,000 (a) and 200,000 (b) nuclei loading inputs. The gray dashed line indicates the threshold (1000 reads) to identify a barcode as a cell. Barcode combinations with fewer than 100 total reads are not plotted. c) The number of nuclei (with a cutoff of 1000 reads) recovered at each loading input. The colors denote the samples multiplexed for each 10X reaction. d,e) Contour plots showing the number of deduplicated reads against the percent of total estimated unique reads that have been observed for each barcode combination at the 100,000 (d) and 200,000 (e) nuclei inputs. The estimated percent of unique reads observed was calculated by dividing the number of observed unique reads by the estimated complexity for each barcode combination. The color legend shows the normalized barcode density (as calculated in ggplot2) scaled from high (yellow) to low (blue). The gray dashed line indicates the threshold to call a cell barcode. The barcode combinations with fewer than 100 total reads are not shown on the plot. f) The estimated collision rate of each mouse sample mixed with human lung cells when loading either 100,000 or 200,000 nuclei. The filled circle and triangle indicate the mouse liver and lung tissues, respectively. The error bar shows the standard error and the black point represents the sample mean at each input. The same samples between the two loading inputs are connected by a gray dashed line. The collision rate reported in each dataset includes doublets derived from the same species. g) Distribution of scaled read depth within a 4000 bp window centered on genome-wide TSSs (with 2000 bp upstream and 2000 bp downstream) by aggregating all cells from lung (upper panels) or liver (lower panels) samples for each technique. The txci-ATAC data (red) combined the libraries of 100,000 and 200,000 loading inputs. X-axis indicates the base position relative to the TSS. Y-axis indicates the read depth for that position summarized across all annotated TSSs. The values are scaled relative to the mean coverage for the 100 bp at either end of this 4000 bp window.


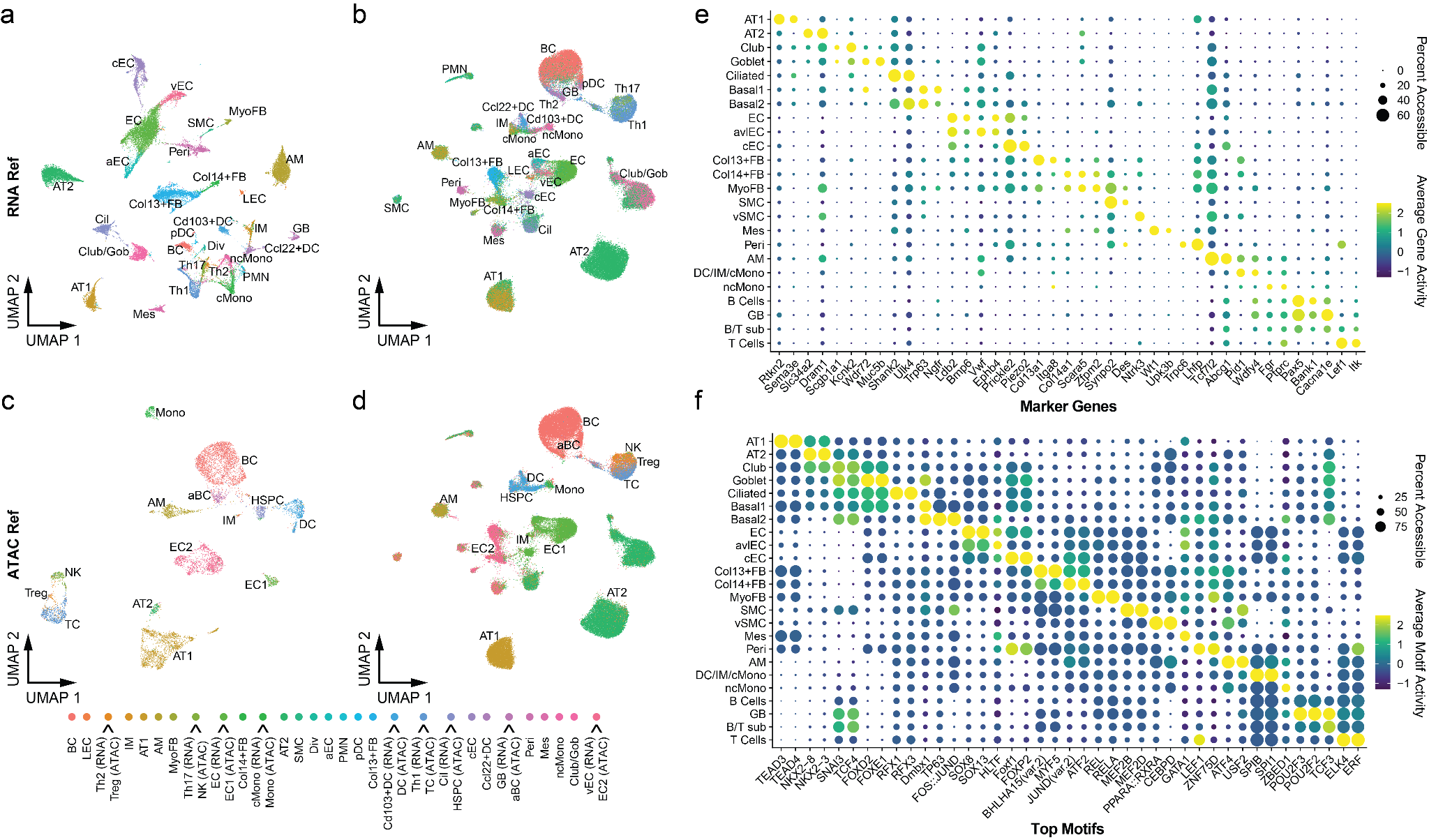


**Fig S6. Cell type annotation of mouse lung samples.** a) UMAP of sc/snRNA-seq reference in which one scRNA-seq sample was integrated with two replicate samples of snRNA-seq, all from the same study. b) UMAP of txci-ATAC-seq data annotated with the labels predicted by the integrated scRNA-seq reference. c) UMAP of the sci-ATAC-seq reference. d) UMAP of txci-ATAC-seq data annotated with the labels predicted by the sci-ATAC-seq reference. The color legend for all panels is shown on the right. The legend labels with the assay enclosed in parentheses (and connected to a color with a line) denote that these cell-type labels are only observed in one reference (“RNA” for the data shown in (a), and “ATAC” for the data shown in (b)) and share a color with a cell type that is only observed in the other reference. Abbreviations: aBC, activated B cells; aEC, arterial endothelial cells; AM, alveolar macrophages; AT1, alveolar type 1 epithelial cells; AT2, alveolar type 2 epithelial cells; BC, B cells; cEC, capillary endothelial cells; Cil, ciliated cells; cMono, classical monocytes; Col13+FB, collagen type XIII α 1 chain positive fibroblasts; Col14+FB, collagen type XIV α 1 chain positive fibroblasts; DC, dendritic cells; Div, dividing cells; EC, endothelial cells; GB, germinal B cells; Gob, goblet cells; HSPC, Hematopoietic progenitors; IM, interstitial macrophages; LEC, lymphatic endothelial cells; Mes, mesothelial cells; MyoFB, myofibroblasts; ncMono, nonclassical monocytes; NK, natural killer cells; pDC, plasmacytoid dendritic cells; Peri, pericytes; PMN, neutrophils; SMC, smooth muscle cells; TC, T cells; Treg, regulatory T cells; vEC, venous endothelial cells. e,f) Dot plots of gene activity (e) and motif activity (f) scores for marker genes and top motifs identified using differential activity tests. One or two maker genes for each cell type were selected for plotting based on the specificity of their gene activity scores and restricted expression patterns observed using online scRNA-seq data browsers [[30,35]](https://paperpile.com/c/lsPqWS/Ycudc+fp7F). The motifs were chosen for plotting among those that were identified as significantly differentially accessible. The dot color depicts the scaled average gene or motif activity scores across all cells within a cell type, while the size of the dot encodes the percentage of cells in that cell type.


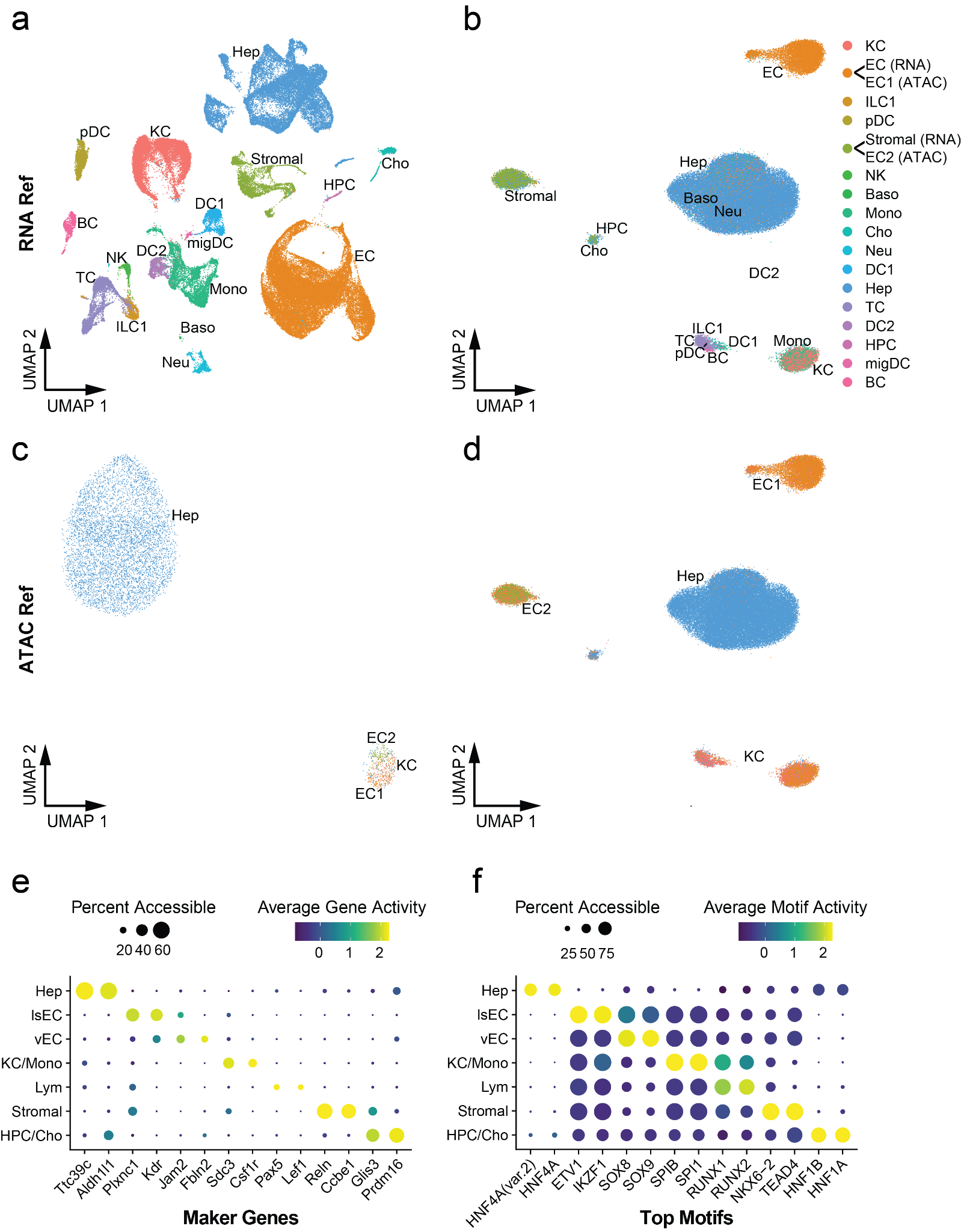


**Fig S7. Cell type annotation of mouse liver samples.** a) UMAP of sc/snRNA-seq reference integrating the snRNA-seq with scRNA-seq using different digestion protocols. b) UMAP of txci-ATAC-seq data annotated with the labels predicted by scRNA-seq reference. c) UMAP of sci-ATAC-seq reference. d) UMAP of txci-ATAC-seq data annotated with the labels predicted by sci-ATAC-seq reference. The color legend for all panels is shown on the right. The legend labels with the assay enclosed in parentheses (and connected to a color with a line) denote that these cell-type labels are only observed in one reference (“RNA” for the data shown in (a), and “ATAC” for the data shown in (b)) and share a color with a cell type that is only observed in the other reference. Abbreviations: Baso, basophils; BC, B cells; Cho, cholangiocytes; DC, conventional dendritic cells; EC, endothelial cells; Hep, hepatocytes; HPC, hepatic progenitor cells; ILC1, type 1 innate lymphoid cells; KC, Kupffer cells; migDC, migratory DCs; Mono, monocytes and monocyte-derived cells; Neu, neutrophils; NK, NK cells; pDC, plasmacytoid dendritic cells; TC, T cells. e,f) Dot plots of gene activity (e) and motif activity (f) scores for marker genes and top motifs identified using differential activity tests. Two maker genes for each cell type were selected for plotting based on the specificity of their gene activity scores and restricted expression patterns observed using an online scRNA-seq data browser [[31]](https://paperpile.com/c/lsPqWS/rApIT). Two motifs were chosen for plotting among the significantly differentially accessible motifs identified for each cell type. The dot color depicts the scaled average gene or motif activity scores across all cells within a cell type, while the size of the dot encodes the percentage of cells in that cell type.


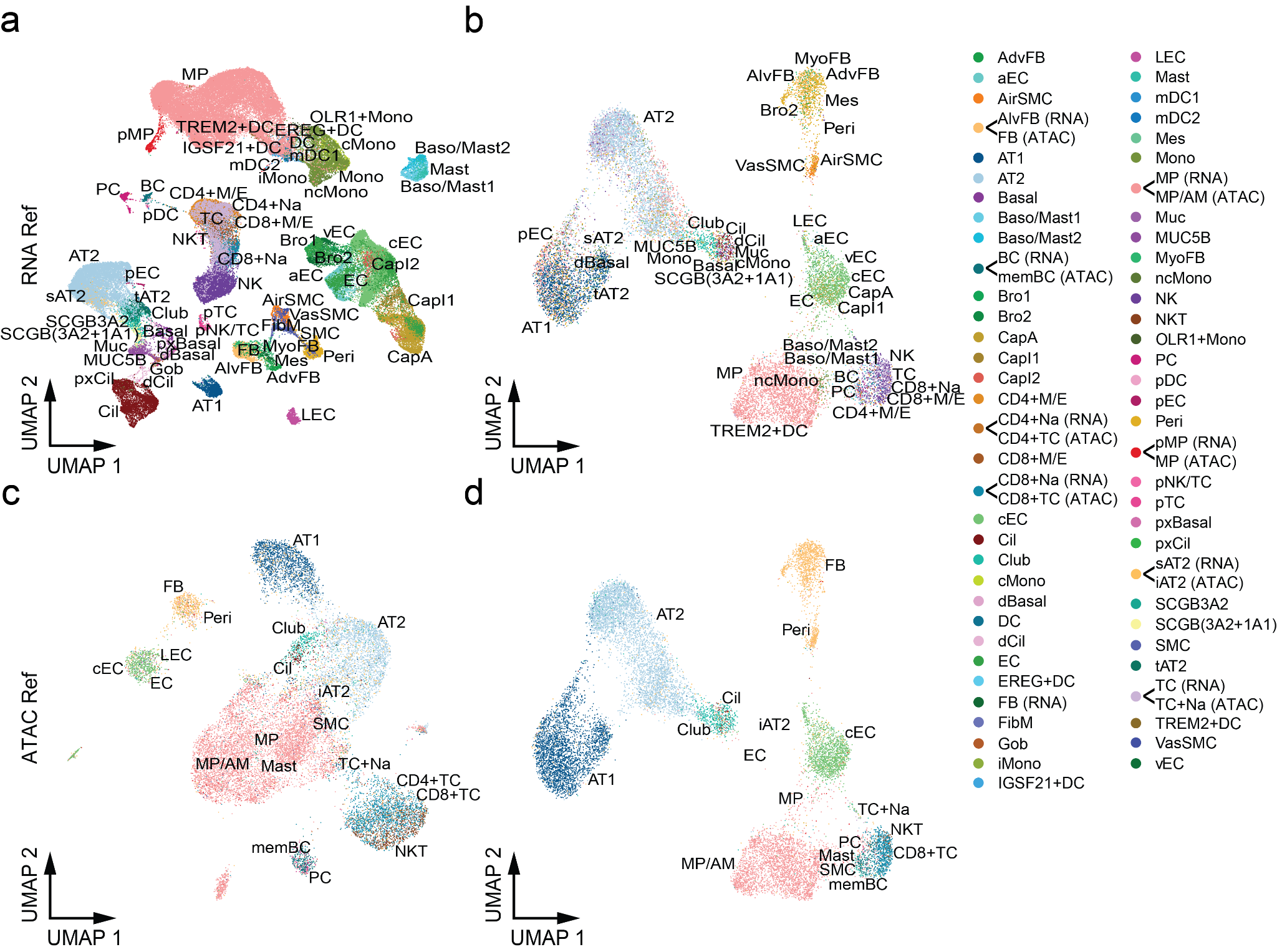


**Fig S8. Cell type annotation of human lung sample with label transfer.** a) UMAP of scRNA-seq reference integrating the two scRNA-seq datasets. b) UMAP of txci-ATAC-seq data annotated with the labels predicted by scRNA-seq reference. c) UMAP of sci-ATAC-seq reference. d) UMAP of txci-ATAC-seq data annotated with the labels predicted by sci-ATAC-seq reference. The color legend for all panels is shown on the right. The legend labels with the assay enclosed in parentheses (and connected to a color with a line) denote that these cell-type labels share a color with a cell type that is observed in the alternative reference (“RNA” for the data shown in (a), and “ATAC” for the data shown in (b)). Note: the fibroblasts (FB) in ATAC reference share the color with alveolar fibroblasts (AlvFB) rather than FB in RNA reference; The macrophages (MP) in RNA reference share the color with general/alveolar macrophages (MP/AM) rather than MP in ATAC reference. Abbreviations: AdvFB, adventitial fibroblasts; aEC, arterial endothelial cells; AirSMC, airway smooth muscle cells; AlvFB, alveolar fibroblasts; AT1, alveolar type 1 epithelial cells; AT2, alveolar type 2 epithelial cells; Baso/Mast1, basophil/mast cell 1 cells; Baso/Mast2, basophil/mast cell 2 cells; BC, B cells; Bro1, bronchial vessel 1 cells; Bro2, bronchial vessel 2 cells; CapA, capillary aerocytes; CapI1, capillary intermediate 1 cells; CapI2, capillary intermediate 2 cells; CD4+M/E, CD4+ memory/effector T cells; CD4+Na, CD4+ naive T cells; CD4+TC, CD4+ T cells; CD8+M/E, CD8+ memory/effector T cells; CD8+Na, CD8+ naive T cells; CD8+TC, CD8+ T cells; cEC, capillary endothelial cells; Cil, ciliated cells; cMono, classical monocytes; dBasal, differentiating basal cells; DC, conventional dendritic cells; dCil, differentiating ciliated cells; EC, endothelial cells; FB, fibroblasts; FibM, fibromyocytes; Gob, goblet cells; iAT2, alveolar type 2/immune; iMono, intermediate monocytes; LEC, lymphatic endothelial cells; mDC1, myeloid dendritic type 1 cells; mDC2, myeloid dendritic type 2 cells; memBC, memory B cells; Mes, mesothelial cells; Mono, monocytes; MP, macrophages; MP/AM, macrophages (general/alveolar); Muc, mucous cells; MUC5B, MUC5B+ secretory cells; MyoFB, myofibroblasts; ncMono, nonclassical monocytes; NK, natural killer cells; NKT, natural killer T cells; PC, plasma cells; pDC, plasmacytoid dendritic cells; pEC, proliferating epithelial cells; Peri, pericytes; pMP, proliferating macrophages; pNK/TC, proliferating NK/T cells; pTC, proliferating T cells; pxBasal, proximal basal cells; pxCil, proximal ciliated cells; sAT2, signaling AT2 cells; SCGB3A2, SCGB3A2+ secretory cells; SCGB(3A2+1A1), SCGB3A2+ and SCGB1A1+ secretory cells; SMC, smooth muscle cells; tAT2, transitional AT2 cells; TC, T cells; TC+Na, naive T cells; VasSMC, vascular smooth muscle cells; vEC, venous endothelial cells.


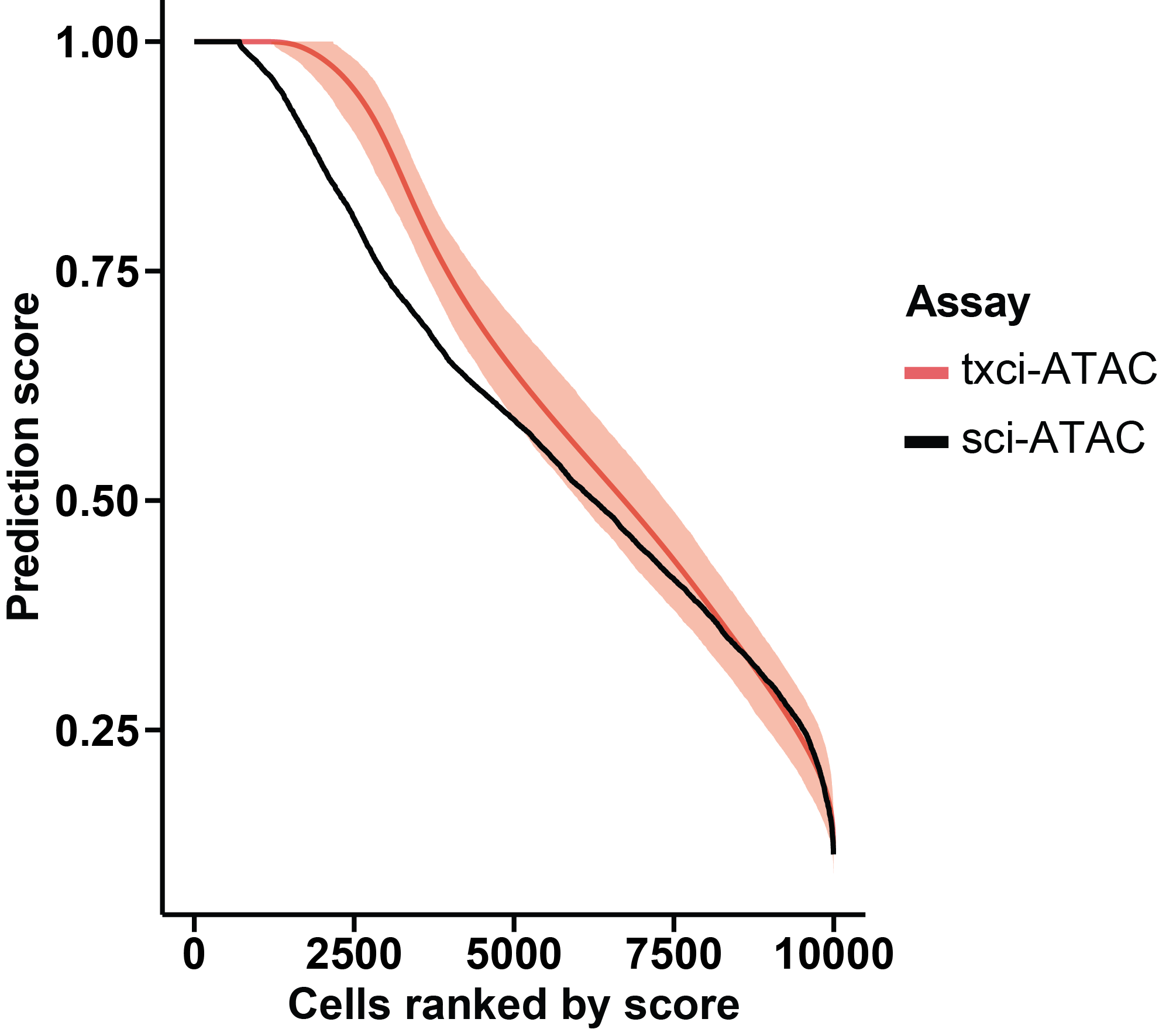


**Fig S9. Comparison of prediction accuracy between txci-ATAC-seq and sci-ATAC-seq in mouse lung cells.** The txci-ATAC-seq dataset was subsampled to have the same number of cells as that in sci-ATAC-seq data 1000 times. The prediction score (y-axis) calculated by Seurat label transfer using an RNA-seq reference was plotted against the cell ranks (x-axis) based on the prediction score. The red line shows the mean score of 1000 simulations in txci-ATAC-seq. The shaded band is the pointwise 95% confidence interval based on subsampling (from the 2.5% to 97.5% quantile). The black line shows the prediction score in sci-ATAC-seq data.


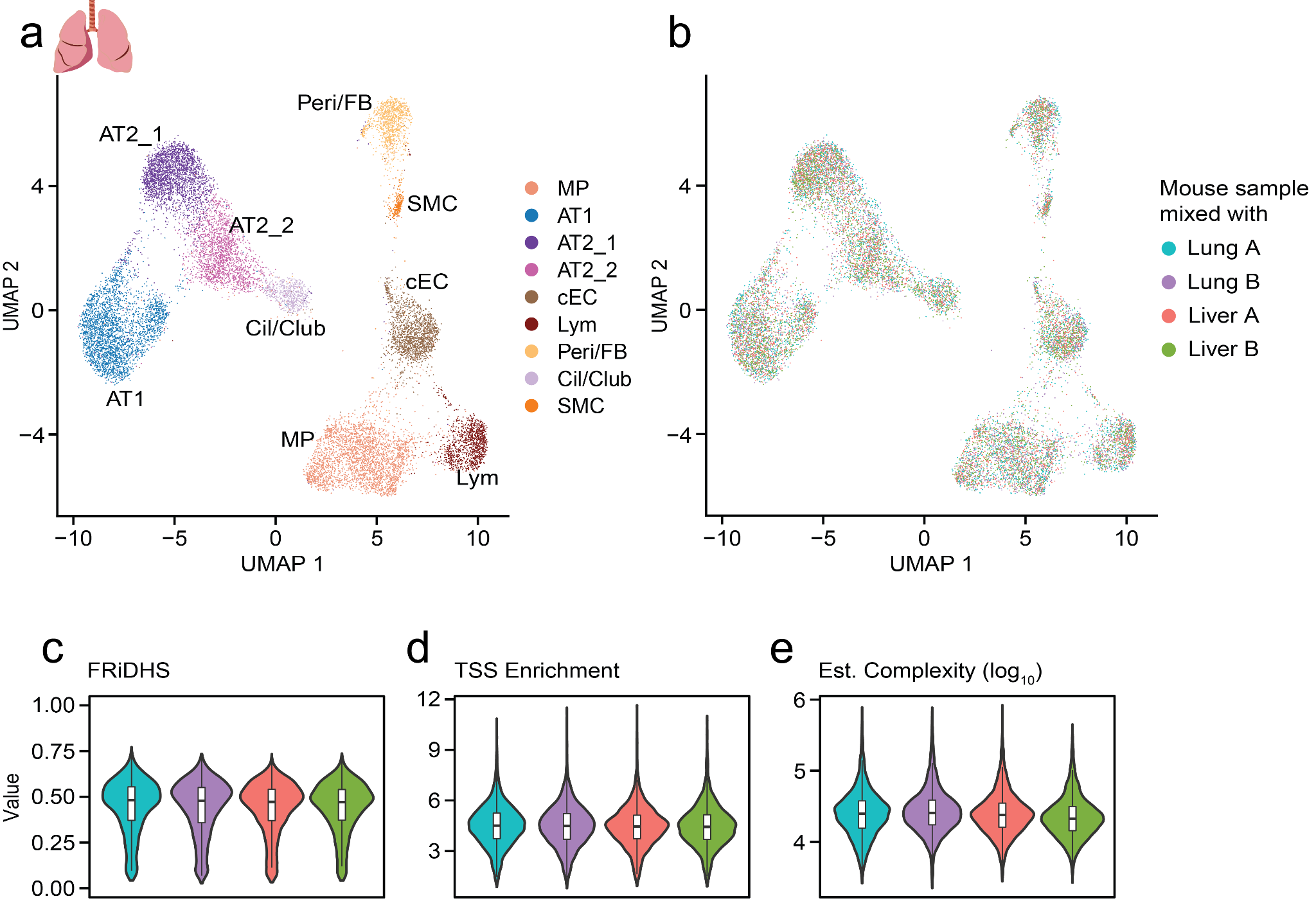


**Fig S10. Characterization of cellular heterogeneity in human lung tissue.** a) UMAP visualization of human lung nuclei (n = 15,799) identifying 9 distinct cell types. See abbreviation of cell labels in Additional file 1: Fig. S8. b) UMAP of human lung nuclei visualized by the mouse samples with which they were mixed. c-e) QC metrics of human lung nuclei mixed with different mouse samples. The color legend is consistent with panel (b). The (c) FRiDHS, (d) TSS enrichment score, and (e) estimated complexity (on a log_10_ scale) are plotted for each human lung nuclei sample profiled across different barnyard settings.


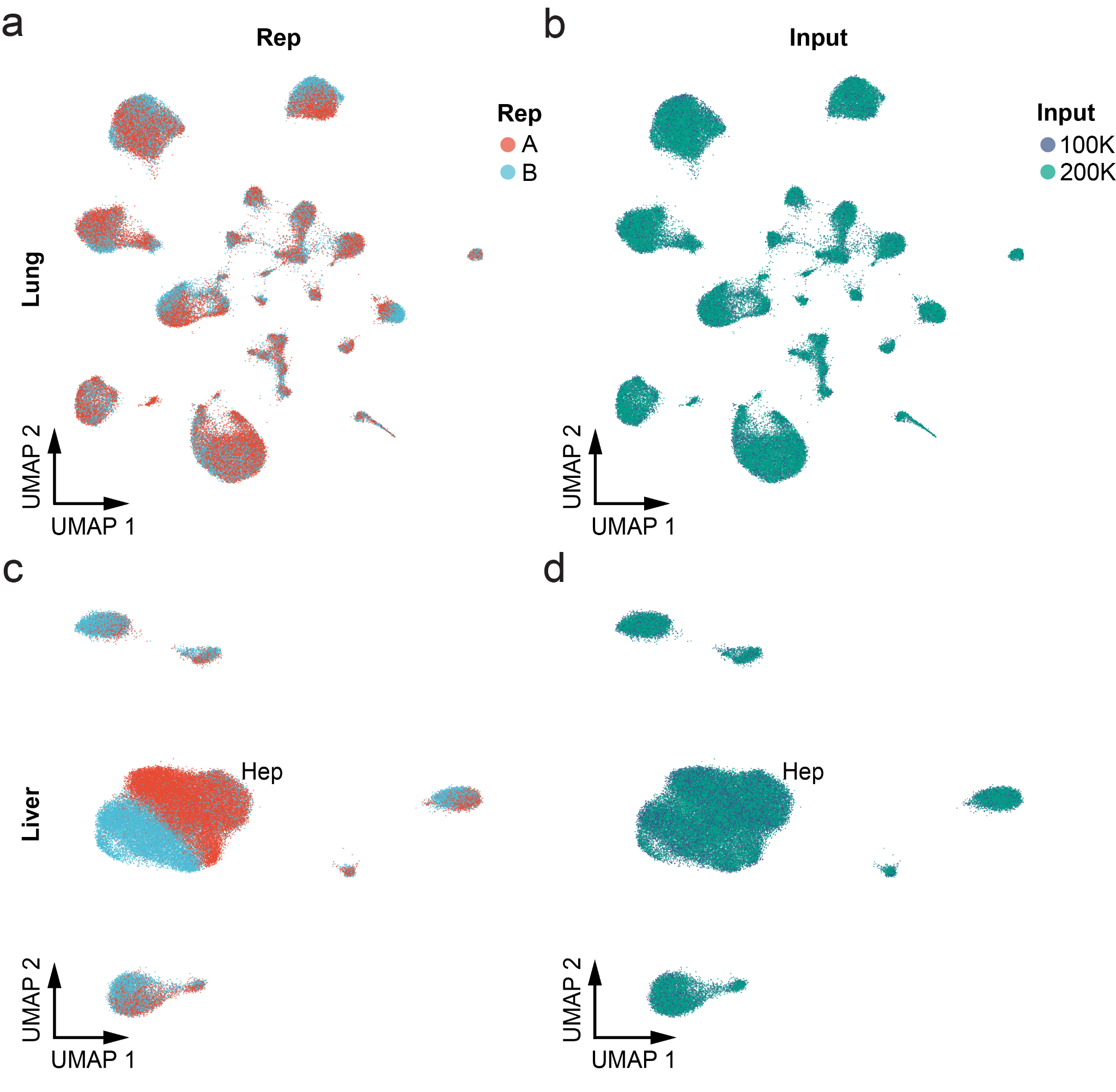


**Fig S11. txci-ATAC-seq are robust to batch effects.** a,b) UMAP visualization of mouse lung samples showing the batch variance introduced either by (a) mouse replicate or (b) nuclei input on the 10X. c,d) UMAP visualization of mouse liver samples showing the batch variance introduced either by (c) mouse replicate or (d) nuclei input. Colors for replicates and inputs are consistent in both (a,c) and (b,d). Hepatocytes are indicated by “Hep” in (c).


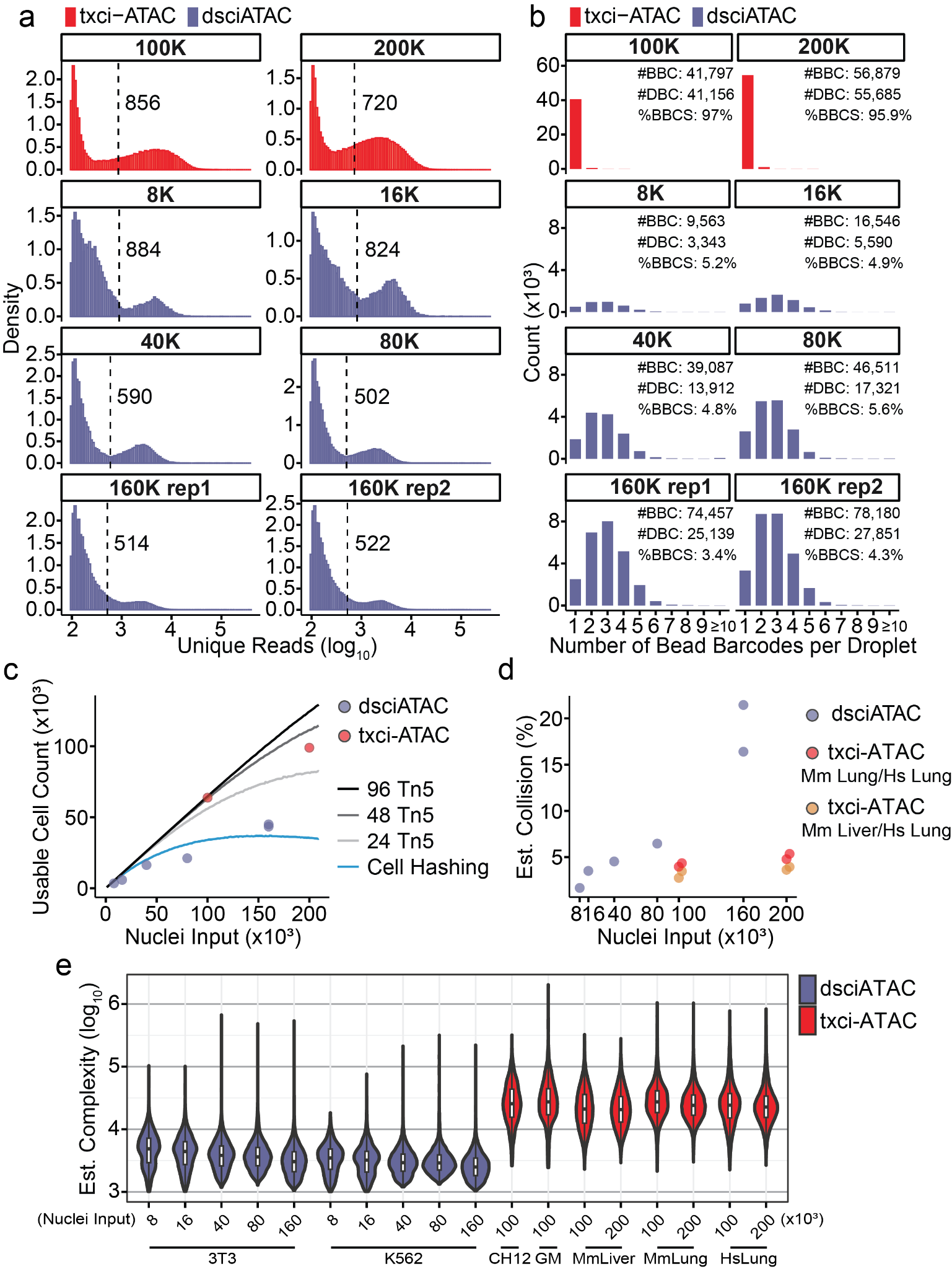


**Fig S12. Comparison of txci-ATAC with dsciATAC using a bead-merging strategy.** a) Distribution of log_10_-transformed unique read counts assigned to each cell barcode (after combining bead barcodes that were present in the same droplet with Tn5 barcodes) in the txci-ATAC (red) or dsciATAC (slate blue) datasets. The dashed line, accompanied by the associated value, indicates the read depth threshold for cells determined by *K*-means clustering. Barcodes with less than 100 reads are excluded from the plot. b) Quantification of the number of bead barcodes predicted to coexist within each droplet across both txci-ATAC (red) and dsciATAC (slate blue) datasets. The x-axis represents the number of bead barcodes identified per droplet. The droplets containing 10 or more bead barcodes were consolidated. The annotations in each panel denote the total number of bead barcodes (#BBC), the total number of droplet barcodes (#DBC) after merging the bead barcodes identified within the same droplets, and the percentage of bead barcodes within the droplets having a single bead barcode (%BBCS). c,d) The number of deconvoluted cells (c) and estimated collision rate (d) calculated from the cells identified using a read depth cutoff of 1,000 reads for both txci-ATAC (using 96 Tn5 barcodes, red dots) and dsciATAC (using 48 Tn5 barcodes, slate blue dots) datasets across different nuclei loading inputs. In panel (c), both the simulated cells recovered by the cell hashing (blue line) and molecular hashing approaches (black and gray lines) were compared with the empirical cell recovery obtained from the txci-ATAC and dsciATAC data. In panel (d), a mixture of human K562 and murine 3T3 cells was used to identify multiplets in dsciATAC datasets (slate blue). For txci-ATAC, the collision cells were examined using either a mixture of mouse and human lung cells (red) or a mixture of mouse liver and human lung cells (orange). The data points from the txci-ATAC datasets were jittered to enhance the visibility of individual replicates. e) Comparison of estimated library complexity between txci-ATAC and dsciATAC at different nuclei loading inputs spanning various samples. The library complexity is displayed on a log_10_ scale. GM: GM12878; MmLiver: Mouse liver; MmLung: Mouse lung; HsLung: Human lung.


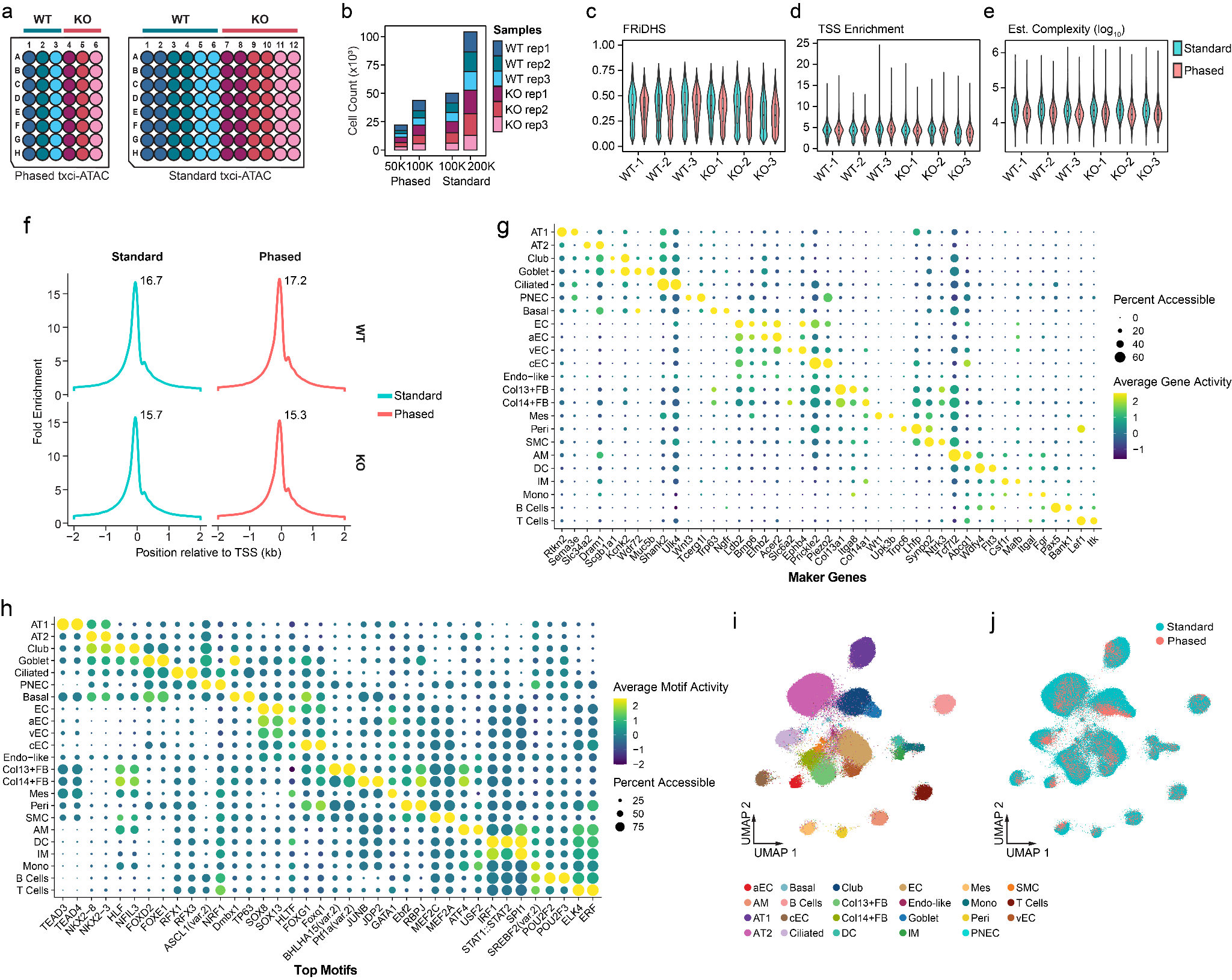


**Fig S13. Phased-txci-ATAC-seq improves multiplexing capability without sacrificing data quality.** a) Well assignment for the phased and standard versions of txci-ATAC-seq multiplexing WT and CC16 knockout lungs with (3 replicate mice for each genotype). b) The number of nuclei passing quality filters for each protocol at different nuclei loading inputs. c-e) The comparison of quality metrics per cell between the two protocols across 6 mouse lung samples. The Phased-txci-ATAC-seq provided a comparable FRiDHS (c) and TSS enrichment score (d) but slightly lower estimated complexity (e) than the standard protocol. f) Distribution of scaled read depth within a 4000 bp window centered on the TSSs (with 2000 bp upstream and 2000 bp downstream) by aggregating all cells from either WT (upper panels) or CC16^-/-^ (lower panels) lung samples for each assay. X-axis indicates the base position relative to the TSS. Y-axis indicates the read depth for that position summarized across all annotated TSSs. The values are scaled relative to the mean coverage for the 100 bp at either end of this 4000 bp window. g,h) Dot plots of gene activity (g) and motif activity (h) scores for marker genes and top motifs identified using differential activity tests. One or two maker genes for each cell type were selected for plotting based on the specificity of their gene activity scores and restricted expression patterns observed using online scRNA-seq data browsers [[30,35]](https://paperpile.com/c/lsPqWS/Ycudc+fp7F). The motifs were chosen for plotting among those that were identified as significantly differentially accessible. The dot color depicts the scaled average gene or motif activity scores across all cells within a cell type, while the size of the dot encodes the percentage of cells in that cell type. i,j) UMAP visualization of nuclei by co-embedding the standard (n=154,103) and phased (n=65,799) assays. The nuclei are colored either by predicted cell type (i) or ATAC-seq protocol (j).


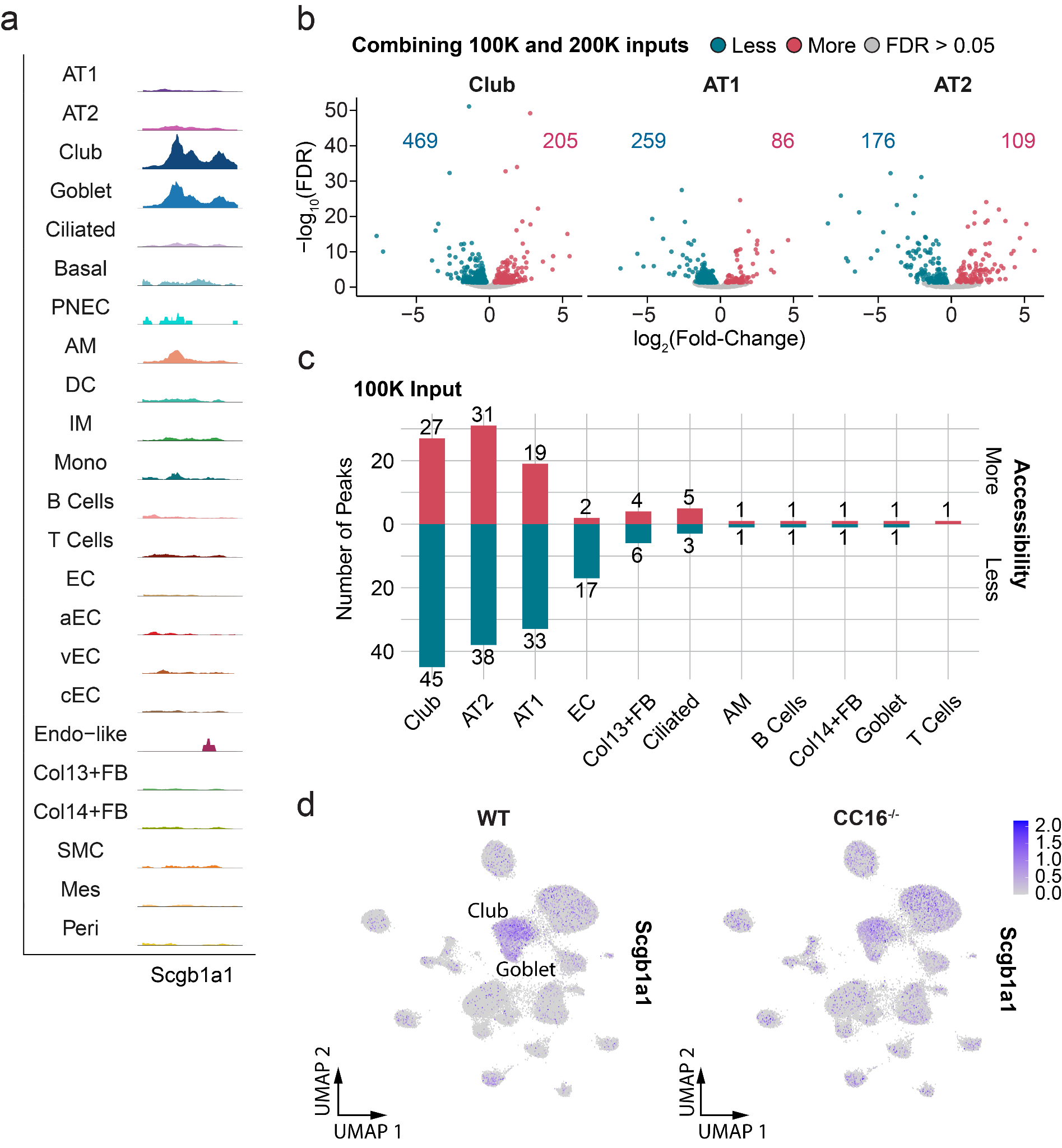


**Fig S14. Chromatin accessibility changes induced by CC16^-/-^ deficiency in mouse lung.** a) Aggregated chromatin accessibility at the *Scgb1a1* promoter region across cell types. The aggregated accessibility signal for each cluster was normalized by a scaling factor computed as the number of cells in the cluster multiplied by the mean sequencing depth for the cells in that cluster. b) Differentially accessible peaks between CC16^-/-^ and WT samples across club, AT1, and AT2 cells generated by combining the 100,000 and 200,000 nuclei loading inputs. The -log_10_-transformed adjusted p-value for each peak was plotted against the log_2_(fold-change). The color labels the peaks that are less accessible (blue), more accessible (red), and unchanged (gray) in knockout samples. The number of differentially accessible peaks identified in each cell type is displayed within the plot. c) The number of differentially accessible peaks per cell type calculated using the 100,000 input alone. With 100,000 nuclei as input, many fewer peaks were identified as differentially accessible. d) UMAP visualization of nuclei showing the gene activity score of *Scgb1a1* in WT (left) and CC16^-/-^ (right) lungs.


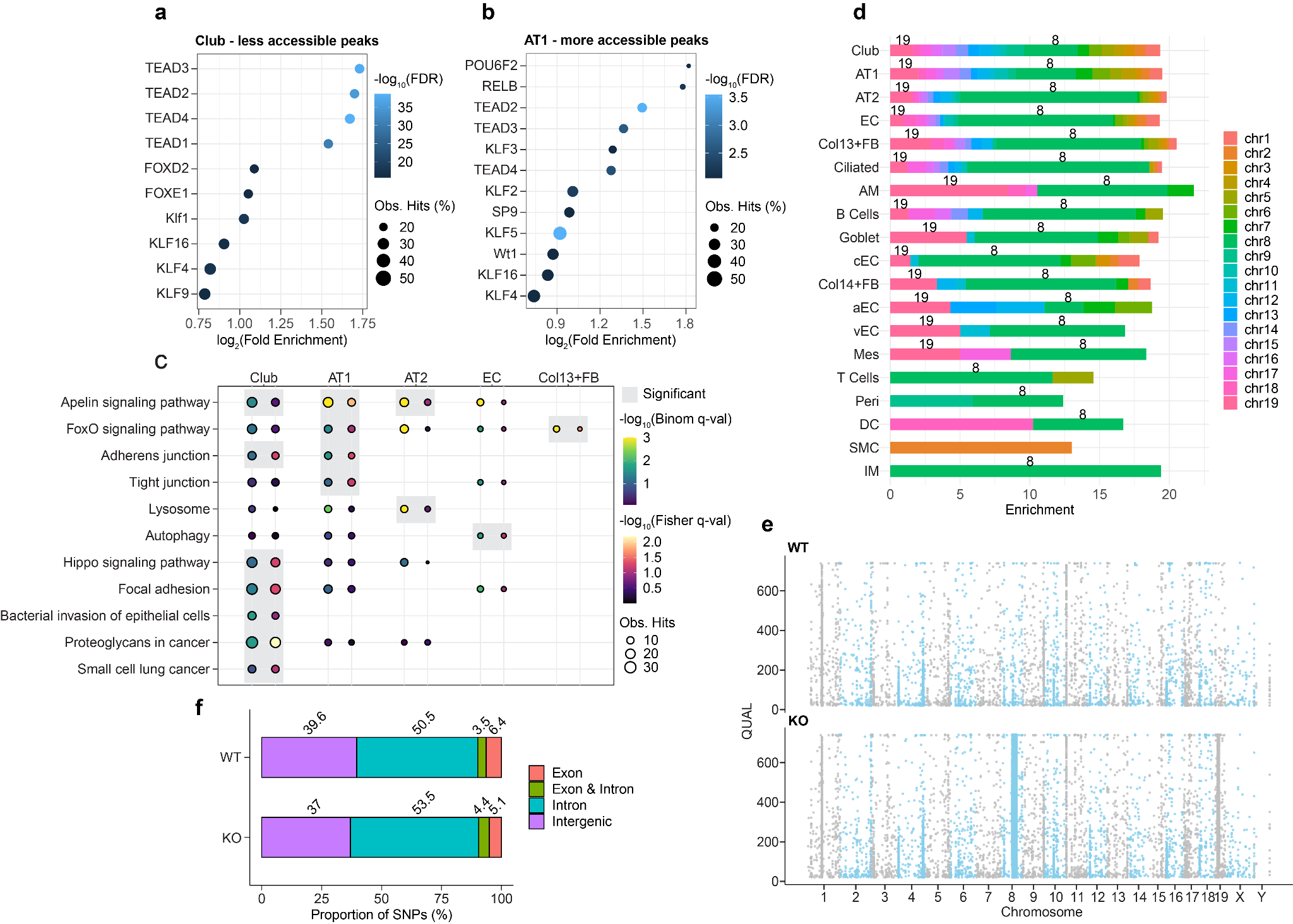


**Fig S15. Functional analysis and regulatory variant identification in CC16 deficient mouse.** a,b) Top 10 motifs significantly enriched in less accessible peaks identified in CC16^-/-^ club cells (a) and more accessible peaks identified in CC16^-/-^ AT1 cells (b). The dot color encodes the -log_10_-transformed adjusted p-value, while the size of the dot encodes the percentage of observed peaks enriched in each motif. The x-axis displays the fold enrichment on a log_2_ scale. c) KEGG pathways of interest enriched in each cell type. The colors show the -log_10_-transformed adjusted p-value derived from the binomial (blue to green) and hypergeometric (black to red) tests. The dot size denotes the number of observed regions (binomial) or genes (hypergeometric) in each test. The significant pathways passing the two-threshold cutoff are highlighted by gray boxes. d) Enrichment of differential peaks in each chromosome across cell types. The enrichment was calculated by dividing the fraction of differential peaks in each chromosome by the fraction of total peaks identified in each chromosome. e) SNVs identified in WT and knockout samples across chromosomes meeting the quality criteria. The y-axis represents the phred-scaled quality score. f) Proportions of SNVs mapped to each functional genomic category (exon, intron, overlapping regions between exon and intron, and intergenic regions) in WT and knockout samples.


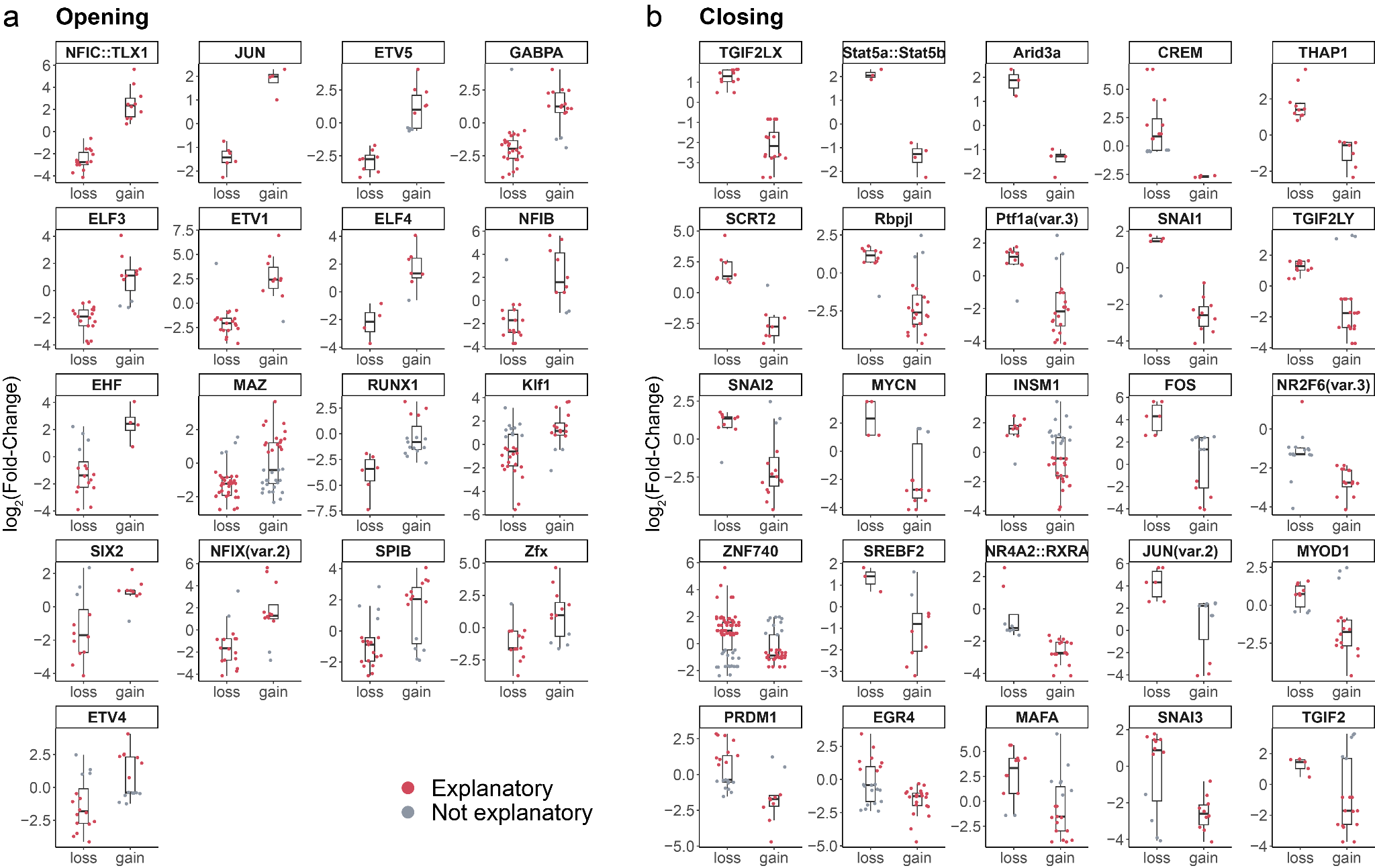


**Fig S16. SNP-driven differences in motif usage alter chromatin accessibility.** a,b) TFs that are associated with increased (a) or decreased (b) chromatin accessibility when the peaks gain the TF motifs. The y-axis shows the log_2_(fold-change) in chromatin accessibility for the differential peaks identified in the SNV hotspots between CC16^-/-^ and WT samples. A positive log_2_(fold-change) means the peaks are more accessible in the knockout samples. The x-axis indicates the motif hits that are gained or lost in the peaks carrying the CC16^-/-^ SNVs. The instances (red) that exhibit a coherent change in chromatin accessibility with the overall motif effect were considered to explain the observed differences in chromatin accessibility between two genotypes (i.e., for opening TF motifs shown in panel (a), the gained instances with a positive log_2_(fold-change) and the lost instances with a negative log_2_(fold-change) were considered explanatory; For closing TF motifs shown in panel (b), the gained instances with a negative log_2_(fold-change) and the lost instances with a positive log_2_(fold-change) were considered explanatory).


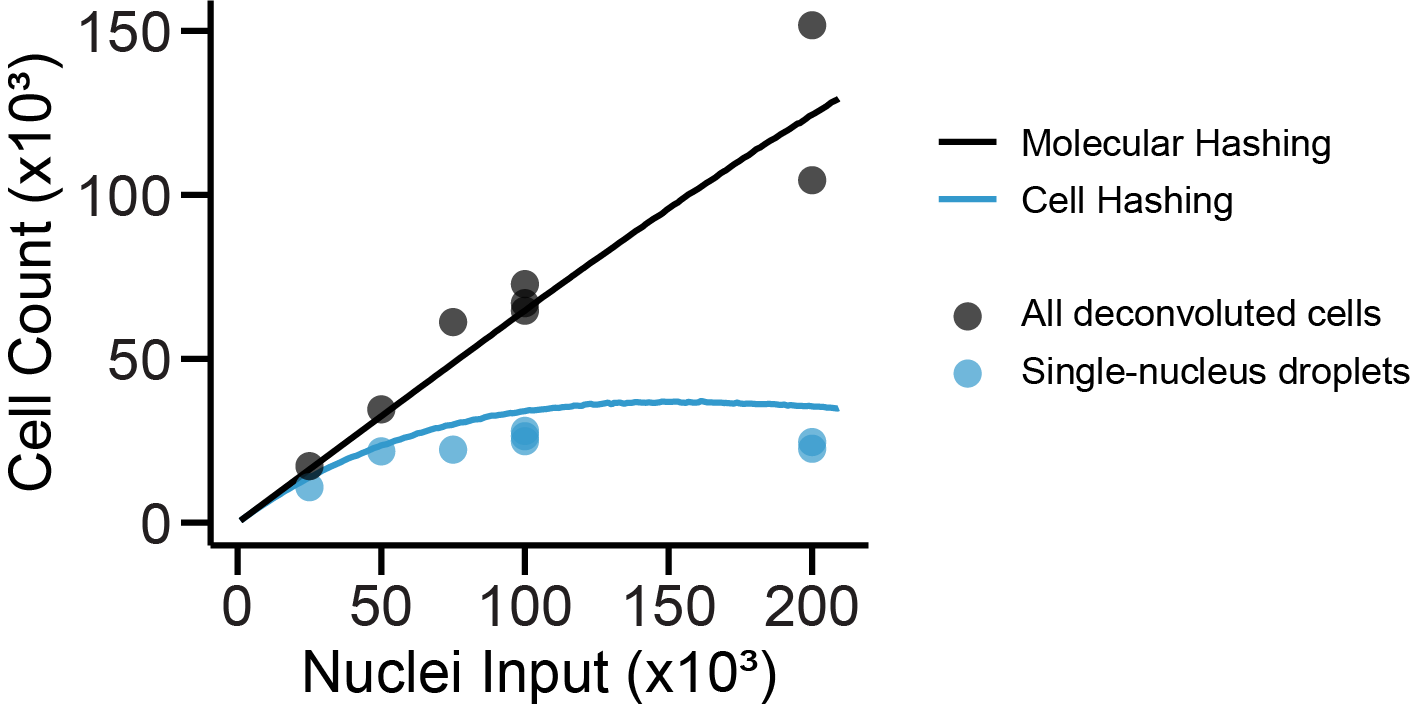


**Fig S17. Comparison of empirical and theoretical cell recovery between molecular and cellular hashing strategies.** The number of cells deconvoluted using molecular indexing (black dots) and cell indexing (blue dots) were compared with the simulated cell recovery obtained from the molecular hashing strategy with 96 Tn5 barcodes (black line) and the cell hashing strategy of discarding all multiplets (blue line).


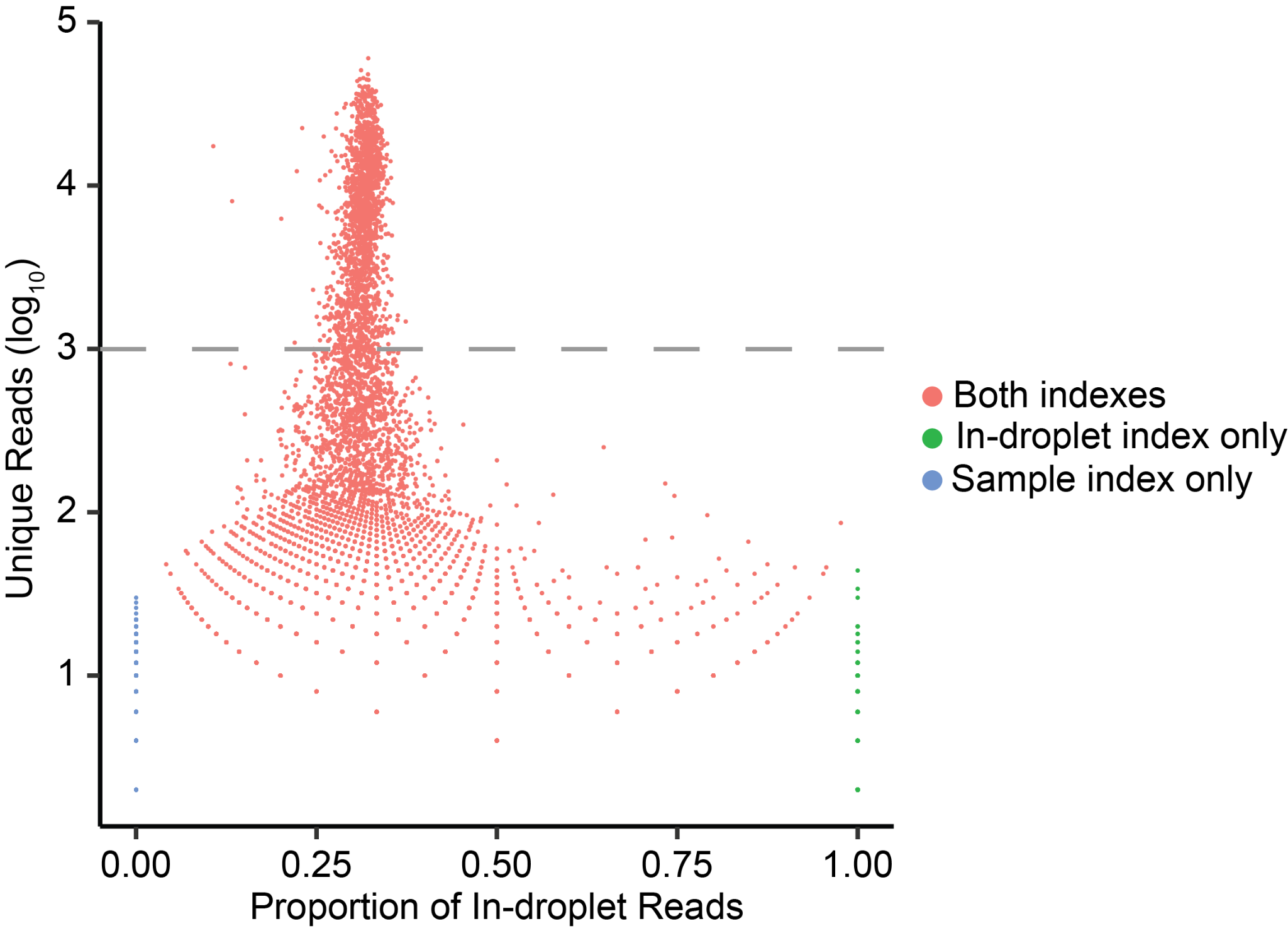


**Fig S18. Examination of efficiency for in-droplet and sample index PCR.** The total unique reads on a log_10_ scale were plotted against the proportion of in-droplet reads for each barcode. The barcodes with both in-droplet and sample indexes were colored in red. The green and blue dots indicate the barcodes only getting the in-droplet index and the barcodes only getting the sample index, respectively. The gray dashed line indicates the threshold to call a cell barcode.
