# Supplementary material for: txci-ATAC-seq: a massive-scale single-cell technique to profile chromatin accessibility
Source: Genome Biol. 2024 Mar 22;25:78. doi: 10.1186/s13059-023-03150-1 (PMC10958877; doi:10.1186/s13059-023-03150-1)
Supplement: Supplementary file 6 — Additional file 6: Review history. [file 13059_2023_3150_MOESM6_ESM.docx]

Review History

**First round of review**

**Reviewer 1**

**Were you able to assess all statistics in the manuscript, including the appropriateness of statistical tests used?**

**Yes:** I am satisfied with statistics used

**Were you able to directly test the methods?**

No

**Comments to author:**

In their manuscript, "txci-ATAC-seq, a massive-scale single-cell technique to profile chromatin accessibility," Zhang, Mulqueen et al present a new single-cell ATAC-seq method that combines 10X barcoding (tx) and single-cell combinatorial indexing (sci) to increase the throughput of standard 10X scATAC experiments. The authors demonstrate that their approach works well on primary human and mouse tissue, and then perform a comparative experiment with wild-type and CC16-/- mice to establish gene regulatory differences in murine lung between these two models. The manuscript is well-written and the method seems robust, though the biological findings are surface-level. This manuscript is highly appropriate for the greater Genome Biology readership and I only have a few comments for the authors to address.

Comments
* The authors do not discuss why end-users might choose to carry out 'fast' txci-ATAC-seq versus standard txsci-ATAC-seq. Is the 'fast' version simply better in all respects? If so, this should be made explicit in the manuscript.

* Please provide an estimate of the cost reduction provided by txsci-ATAC-seq compared to hashing, standard 10X ATAC-seq, or a commercial solution such as Scale Bio.

* To maximize use of the datasets generated here, the authors should host and / or format their data for easy-browsing by readers using one of the available interactive data analysis frameworks for atlas browsing.

**Reviewer 2**

**Were you able to assess all statistics in the manuscript, including the appropriateness of statistical tests used?**

No

**Were you able to directly test the methods?**

No

**Comments to author:**

Genome Biology
txci-ATAC-seq, a massive-scale single-cell technique to profile chromatin accessibility
Hao Zhang et al.

The authors have modified an optimized a single-nuclei pre-indexing approach to be compatible with the 10X genomics platform downstream, for the purpose of achieving higher throughput compared to a standard 10X ATAC-seq workflow while preserving data quality. They tested their workflow, called txci-ATAC-seq, on several different sample types to demonstrate the validity of the method.

From the Genome Biology website description, "Method articles should describe novel methods that are shown to be a clear advance over existing state-of-the-art methods in a side-by-side demonstration, where possible." Overall, I do not deny the potential usefulness of this protocol for those researchers who wish to scale up snATAC-seq studies, but that only have access to the 10X platform (rather than the one previously reported by Lareau et al which uses the BioRad platform). However, I also feel that ultimately the innovation/novelty of this method, as well as the biological significance of the data reported herein, are limited. The general strategy of using pre-indexing has been reduced to practice previously by multiple groups, a point that the authors also discuss in their introduction section. Thus, the main novelty of this manuscript is to adapt this strategy to a different commercial platform. There is also some added value in the optimization of the SBS probes used to reduce Tn5 barcode swapping, but this is a technical point that I feel is not significant enough to justify publication in the current format. I am also uncertain that the data presented shows any clear advance over existing state-of-the-art methods. It is at best comparable to other published methods; but generally speaking, the level of benchmarking done in this manuscript is also insufficient, in terms of methods used for comparison and depth/thoroughness of analysis. The authors can refer to other benchmarking studies to see what types of analyses can and should be done to demonstrate performance. In terms of throughput, there are other methods that achieve much higher throughput.

As for the biological tissues profiled, some of these tissue types have previously been characterized/atlased in other publications (e.g., Duong et al, Cell genomics). The dataset using the CC16-/- mouse is potentially interesting, but the biological insights demonstrated are not validated by any further experimentation, thereby detracting from the significance of the study.

In my view, this manuscript can and should be published somewhere as it could be a useful method for some researchers, but it is not demonstrated to be a substantial improvement over existing methods and therefore doesn't quite fit the scope of Genome Biology Methods in my opinion; it may be more suitable as a protocols publication in a different journal. Or, if the authors generate significantly more biologically interesting analyses, it could become suitable.

In addition to the above comments, I also have some specific points for the authors' consideration:

Page 8 line 161 and line 172 - Lung barnyard and fragment size change after PCR data not shown - Is this an accepted practice at Genome Biology? For data that is very relevant to the study being described, I would like for it to be included in the SI at least. If there is some reason to not include it, please justify. If it is not important to the study, then it should be removed. To me it seems both these two pieces of data are interesting and relevant, and should be included in the SI.

For barnyard experiments, it is stated in the Methods section that they might also include the {multiplet single-species} cases, and that the collision events may be under-estimated as they would not include the multiplets that contain two nuclei from the same species. First, it would be helpful to be clear about this somewhere in figures/figure captions/in the text description of the results, rather than only in the Methods. Second, I am confused why the authors don't also calculate the estimated collision rate based on their barcoding, as a comparison to the modeling-based result. In addition, for mixed species cases, the authors could check for potential mis-barcoding using the species mapping result cross-checked against their barcoding demux result.


Fig S2g shows some comparisons with other methods - would be helpful to include reference citations to those methods in the labels or elsewhere, as the names of each method may not be immediately recognizable to the reader.


Fig 2c - motif images on the right side are too small and hard to read. Suggest moving to SI, or enlarging the figure somehow…

Fig 3c and 3d - numbers on top of the bars overlap with graph lines and make it hard to read… also noted that Lareau et al.'s paper names their method that uses transpositional barcording dsciATAC-seq, not dscATAC which refers only to the use of droplets without overloaded barcoding approach. It would be important to clarify whether the comparison here is done using their combinatorial approach (dsci) or the regular approach (dsc), since the more comparable one would be dsci and not dsc, given that both are using barcoding to achieve overloading. In addition, I think it is important to see a more nuanced comparison of the data to other methods. For example, it is quite standard to perform correlation analyses to show the accuracy/precision when benchmarking against other methods (similar to Fig S1D and S1E in the SNuBAR paper by Kaile Wang et al, or Fig 1C and 1D in the dsciATAC paper by Lareau et al.).

Fig 3e - the result of the data integration appears unsatisfactory. For one, some of the clusters show these tentacle/splatter like features, which are indicative of bad clustering or bad data quality. Since this UMAP is not labelled with cell-type labels, it is hard to say which cluster is which cell type. But given the result shown in Fig 3a, and also the typical biology of the mouse brain, it is surprising that there are so many interconnected clusters (e.g. top left of the UMAP) in Fig 3e when this is not the case in Fig 1a. Furthermore, most clusters show poor integration of different datasets with data points being undermixed, and it is hard to tell if this is a data quality/fragments capture issue due to the methodology, or if it is an integration issue. In our own experience with RNA-seq data, we often experienced poor integration using Harmony on datasets generated by different methods/research groups); the performance is generally better for datasets with less batch effects such as biological replicates using the same method. While I understand that the purpose of this particular integration exercise is not necessarily to integrate these datasets perfectly, it should show that the authors' methods generate data that is comparable to other published methods/datasets. With such poor integration and the cell type labels missing, it is hard to judge whether this is indeed the case. Again, is the poor mixing of the integrated result due to systemic biases in the data itself, or an artefact of the integration algorithm? If the former, then some discussion of where the bias may arises would be important. If the latter, then it should be addressed before publication. There are many integration algorithms published, specifically for ATAC-seq data (note that Harmony is not - it is a general algorithm that CAN be used for ATAC but is not specifically designed for this data type). The authors may get better results with a more ATAC-specific tool.

Figs 4 and 5 - the analysis here is missing some of the standard analyses typically used to evaluate/gain insight from ATAC-seq data, such as the marker gene accessibility landscape, signal enrichment around TSS, and motif analysis (which was done for the brain sample in Fig 2, but not done for the liver and lung).

Fig 5 - for comparison between wt and -/- data, the authors might consider utilizing published bulk data or scRNA-seq data to gain more insight into the gene regulatory landscape caused by -/-, and whether these changes at the chromatin level that they observe can explain the RNA-seq observations.

**Authors Response**

**Point-by-point responses to the reviewers’ comments:**

Authors’ Point-by-Point Response to Reviewers’ Comments

We thank the Reviewers and Editor for the detailed consideration of our manuscript. We found the comments insightful and helpful. We have carefully addressed each comment and genuinely believe that it has resulted in an improved manuscript. Below we replicate the reviewers’ comments in their entirety (in black) along with our responses to each point (in blue). Along with this document, we are submitting a revised manuscript (with changes marked in blue). In addition, addressing the reviewers’ comments necessitated an additional experiment, 12 new figure panels and modifications to 11 existing figure panels, two new supplementary figures, and substantial improvement in the text of the manuscript. The specifics of these changes are elaborated upon in the responses provided below. Furthermore, we noted that the original manuscript lacked a detailed recipe for the loading buffer, so we have included the components we used to make the loading buffer in the revised manuscript. We have also added the well IDs for Tn5 barcodes associated with the WT and CC16 knockout lungs in Table S6 and modified the text accordingly.

Comment from Editor:

In particular given that one of the major proposed points of novelty is the increased ability to super-load one individual well on the 10X platform with a single biological sample (compared to genetic demultiplexing strategies) and recover a higher number of useable droplets, the inclusion of more analysis to compare the throughput of high quality nuclei recovered between different technologies and assays would be a valuable addition and strengthen a revised manuscript considerably.

We thank the editor for their valuable suggestions. We have now considerably expanded our comparisons with existing techniques. This topic is relevant to comments from both of the reviewers. To avoid redundancy, we detail those results here, though we have tried to reference this section where it is relevant in our responses to the reviewers as well below. To explore the theoretically usable droplets generated by both cell hashing methods (like SNuBar and CITE-seq) and Tn5-based molecular hashing methods (like dsciATAC-seq and our txci-ATAC-seq), we simulated the potential recovery of singlets achieved through these strategies and also evaluated the multiplexing capability for molecular hashing methods at different numbers of Tn5 barcodes. For the simulations, inclusion of cells and beads in droplets were modeled under a Poisson distribution with the following assumptions:

1) A total of 100,000 bead-filled droplets can be generated by the 10x Genomics platform based on the observed mean nuclei per droplet.
2) 35% of cells are lost during GEM formation, attributable to the factors such as the dead volume, cell bursting, and encapsulation into empty droplets. This is consistent with the guidelines provided by 10X in their standard protocols.
3) For the molecular hashing approach, which can salvage data from droplets containing more than one nucleus, we assumed that droplets with an expected collision rate greater than 10% would not be retained (resulting in the droplets containing more than 4 cells being discarded for 96-plex Tn5 simulations, those containing more than 3 cells being discarded in 48-plex, and those containing more than 2 cells in 24-plex being discarded).

As the results show below, cell hashing (blue solid line) can generate a maximum of 37,134 singlets when loading ~161,292 nuclei in a single lane of a 10X instrument. However, with 96 Tn5 barcodes (long-dashed line), the singlet recovery of molecular hashing peaked at the 470,680 nuclei input, resulting in a total of 194,970 usable cells, which is ~5-fold increase in cell recovery compared to cell hashing.

While cell hashing allows for super-loading of cells/nuclei, we noticed that the increased throughput is at the expense of sequencing a substantial number of multiplets that cannot be demultiplexed with cellular hashing strategies and therefore need to be discarded. In the simulation below, we evaluated the ratio of unusable cells (from multiplets) to demultiplexable cells for each hashing strategy (between 1,000 and 210,000 nuclei inputs) and found that the ratio exhibited a significantly faster rate of increase in cell hashing compared to molecular hashing methods. This demonstrates that the cost of sequencing per usable cell is substantially higher for cellular hashing strategies.

To address points raised by the Editor and Reviewers, we used our analysis pipeline to reanalyze the data produced by the dsciATAC, a Tn5-based molecular hashing approach implemented on the Bio-Rad single-cell platform for a clearer comparison. We compared the singlet recovery between dsciATAC and our txci-ATAC. In the dsciATAC paper, they also overloaded bead barcodes to boost library complexity. To make a fair comparison, we inferred the bead multiplets (which refers to the droplets containing more than one bead barcode) for both dsciATAC and txci-ATAC datasets using the bead-based scATAC processing (bap) package as described in the dsciATAC paper and merged the bead barcodes present in a common droplet followed by read deduplication and detection of collision cells. Both datasets underwent the same process. To achieve a robust conclusion, we evaluated these two techniques using two different read depth thresholds for defining cells: 1) a universal cutoff of 1,000 reads across all datasets (Fig S4j,k), and 2) an automatically identified cutoff using *K*-means clustering (Fig 4g,i). The results consistently demonstrated that our txci-ATAC outperformed dsciATAC in both cell recovery and collision rate, regardless of approaches used for cell threshold determination.

In addition, in the dsciATAC paper, the authors noted a lower library complexity, which they attributed to the use of suboptimal Tn5 concentration, but the results were not shown in the paper. After reanalysis of their data, we found that their estimated library complexity for different experiments had a median that ranged from 2,690 to 4,054, which is much lower than the complexity observed in our txci-ATAC data (a median of 22,739 to 24,493). It is undetermined at this point if the low complexity issue for dsciATAC can be solved by simply tweaking the Tn5 concentration.

We have incorporated the following text within the result and method sections and added associated figures in the manuscript to elucidate the simulation results and comparison of QC metrics between dsciATAC and txci-ATAC.

From page 17 line 339 to page 19 line 378, we have added the following text:

“To gain insight into the improvement of Tn5-based molecular hashing approaches (such as our txci-ATAC-seq) in cell recovery as compared to the cellular hashing methods, we simulated the cell recovery outcome as a function of loading input spanning from 1,000 to 1.5 million nuclei for both hashing strategies, modeling cells in droplets under a Poisson distribution (See Methods). The theoretical estimates of cellular hashing were evaluated using the cell recovery reported in the SNuBar paper (Fig. 4g). Similarly, the evaluations of molecular hashing estimates using different numbers of Tn5 barcodes, were conducted by comparing them with the cell recovery measured in both txci-ATAC (96 Tn5 barcodes) and dsciATAC (48 Tn5 barcodes) datasets across a range of input nuclei [9]. Because a bead overloading strategy was employed for the dsciATAC method, we identified bead multiplets (which refers to droplets containing more than one bead barcode) in both droplet-based assays using the bead-based ATAC processing (bap) package [40] and then merged the bead barcodes inferred to have been present in the same droplet prior to downstream analysis (see Methods). The cell thresholds were automatically determined by performing *K*-means clustering on log_10_-transformed read counts for each dataset (Fig. S12a). While it had been previously reported that ~13-21% of bead barcodes could be derived from multi-bead droplets for the 10X platform [40], we only inferred ~3-4.1% of bead barcodes affected by bead multiplets in our txci-ATAC system (Fig. S12b), suggesting that this phenomenon has a minor impact on our txci-ATAC data. Comparing molecular and cellular hashing strategies, the theoretical maximum of cells recovered that can be deconvoluted by molecular hashing was ~5 times higher than that achieved by cell hashing (Fig. 4g). When using 96 barcodes for molecular hashing (as demonstrated for our txci-ATAC-seq method), the number of deconvoluted cells from a single lane of the 10X instrument was maximized at an input of 470,680 total nuclei, yielding data from 194,970 usable cells. Cellular hashing, however, only reached a maximum of 37,134 usable cells upon loading 161,292 nuclei. Notably, txci-ATAC-seq empirical data not only achieved a substantially higher cell recovery rate than dsciATAC (averaging 61.0% in txci-ATAC vs. 39.1% in dsciATAC), but also exhibited a closer alignment with the theoretical expectations (Fig. 4g). A further evaluation of the ratio of collision cells (i.e. cells that could not be deconvoluted) to usable cells through simulation revealed that the increased throughput in the cell hashing also requires the added cost of sequencing a substantially higher number of unusable cells than molecular hashing strategies (Fig. 4h). Comparing the two molecular hashing strategies head-to-head, txci-ATAC-seq consistently demonstrated lower collision rates than dsciATAC (Fig. 4i). The improved performance of txci-ATAC in both cell recovery and collision rate reduction remained evident even using a simple universal read depth threshold for determining cells (Fig. S12c,d). In addition, a significantly higher library complexity was observed for txci-ATAC compared to that obtained by dsciATAC (Fig. S12e). While this may be partially explained by differences in sample types, the lower complexity measured in dsciATAC could be attributed to the fundamental difference in single-cell platforms employed by the txci-ATAC and dsciATAC as a lower recovery of unique fragments was reported in the Bio-Rad system compared to the 10X [10].”

Fig. S12. **Comparison of txci-ATAC with dsciATAC using a bead-merging strategy.** a) Distribution of log_10_-transformed unique read counts assigned to each cell barcode (after combining bead barcodes that were present in the same droplet with Tn5 barcodes) in the txci-ATAC (red) or dsciATAC (slate blue) datasets. The dashed line, accompanied by the associated value, indicates the read depth threshold for cells determined by *K*-means clustering. Barcodes with less than 100 reads are excluded from the plot. b) Quantification of the number of bead barcodes predicted to coexist within each droplet across both txci-ATAC (red) and dsciATAC (slate blue) datasets. The x-axis represents the number of bead barcodes identified per droplet. The droplets containing 10 or more bead barcodes were consolidated. The annotations in each panel denote the total number of bead barcodes (#BBC), the total number of droplet barcodes (#DBC) after merging the bead barcodes identified within the same droplets, and the percentage of bead barcodes within the droplets having a single bead barcode (%BBCS).

Fig. 4g-i. g) Theoretical and empirical number of deconvolutable cells recovered across various nuclei loading inputs for molecular and cellular hashing strategies. The simulated cell recovery for either molecular hashing at different numbers of Tn5 barcodes (black and gray lines) or cellular hashing (blue line) strategies were compared with the observed cell recovery obtained from the txci-ATAC (indexed with 96 Tn5 barcodes, red dots), dsciATAC (indexed with 48 Tn5 barcodes, slate blue dots), and SNuBar (blue dots) datasets. For txci-ATAC and dsciATAC, the data were processed using the same pipeline, and the cell threshold was determined using *K*-means clustering. For SNuBar, the recovery values were taken directly from the original paper. The lower panel zooms in on the range highlighted by the dashed box in the upper panel. h) Unusable-to-usable cell ratio derived from the simulated data presented in panel (g). Unusable refers to cells that had to be discarded because they were in unresolvable multiplets. Usable cells include singlets and cells that can be unambiguously demultiplexed from multiplets for molecular hashing strategies. The colors are consistent with panel (g). i) Estimated collision rate calculated from the cells determined by *K*-means clustering for both txci-ATAC and dsciATAC datasets across various loading inputs. A mixture of human K562 and murine 3T3 cells was used to identify multiplets in the dsciATAC datasets (slate blue). For txci-ATAC, the collision rate was evaluated using either a mixture of mouse and human lung cells (red) or a mixture of mouse liver and human lung cells (orange). The data points from the txci-ATAC datasets were jittered to enhance the visibility of individual values.

Fig. S12. c,d) The number of deconvoluted cells (c) and estimated collision rate (d) calculated from the cells identified using a read depth cutoff of 1,000 reads for both txci-ATAC (using 96 Tn5 barcodes, red dots) and dsciATAC (using 48 Tn5 barcodes, slate blue dots) datasets across different nuclei loading inputs. In panel (c), both the simulated cells recovered by the cell hashing (blue line) and molecular hashing approaches (black and gray lines) were compared with the empirical cell recovery obtained from the txci-ATAC and dsciATAC data. In panel (d), a mixture of human K562 and murine 3T3 cells was used to identify multiplets in dsciATAC datasets (slate blue). For txci-ATAC, the collision cells were examined using either a mixture of mouse and human lung cells (red) or a mixture of mouse liver and human lung cells (orange). The data points from the txci-ATAC datasets were jittered to enhance the visibility of individual replicates. e) Comparison of estimated library complexity between txci-ATAC and dsciATAC at different nuclei loading inputs spanning various samples. The library complexity is displayed on a log_10_ scale. GM: GM12878; MmLiver: Mouse liver; MmLung: Mouse lung; HsLung: Human lung.

We have also modified the following text in the Discussion section (page 28 line 566 to page 29 line 577): and updated Fig. S17 (the original figure ID is Fig. S15).

“By quantifying the number of hashing barcodes in each droplet, we can empirically assess cell recovery in both cellular and molecular indexing strategies using our txci-ATAC-seq datasets alone. As expected, deconvoluting cells based on molecular indexing exhibited markedly higher cell recovery compared to the cellular indexing deconvolution strategy in which multiplets must be discarded, especially with increasing loading inputs (Fig. S17). In addition, compared to the theoretical cell recovery, the total cells deconvoluted by txci-ATAC aligned closely with the simulated cell recovery achieved through the molecular hashing strategy. However, for cellular hashing, a noticeable reduction in the number of single-Tn5 droplets was observed at the larger loading inputs compared to the theoretical recovery, suggesting that the cellular hashing recovery rate would be more adversely affected than molecular hashing by multiplet rates that exceed our expectations.”

Fig. S17. **Comparison of empirical and theoretical cell recovery between molecular and cellular hashing strategies.** The number of cells deconvoluted using molecular indexing (black dots) and cell indexing (blue dots) were compared with the simulated cell recovery obtained from the molecular hashing strategy with 96 Tn5 barcodes (black line) and the cell hashing strategy of discarding all multiplets (blue line).

We have added the citation of the bap package to the “Data processing and analysis” section on page 46 line 1016 and added the following text to the Methods (page 57 line 1302 to page 59 line 1350):

**“Simulation of cell recovery**

The cell recovery was modeled using the Poisson distribution with the assumption that 1) a total of 100,000 bead-containing droplets were generated by the 10X microfluidic system based on the observed mean nuclei per droplet and 2) a 35% cell loss occurs, attributable to the factors such as the dead volume, cell bursting, and encapsulation into empty droplets (consistent with guidelines provided by the 10X Chromium Next GEM Single Cell ATAC protocol (v1.1, Document No. CG000209 Rev D). We simulated the nuclei loading inputs ranging from 1,000 to 1.5 million. The mean number of nuclei per droplet (𝜆) was defined as the following equation:

$\lambda=\frac{N}{100,000}\times0.65$, in which *N* is the number of nuclei loading input.

The collision rate in a given droplet was defined as the probability of at least one duplicate barcode present in that droplet (P), and was calculated with the following equation:

$$P=1-\frac{m \times(m-1) \times(m-2) \ldots\times(m-n+1)}{m^{n}} if n\leq m$$

$$P=1 if n>m$$

The m represents the number of Tn5 barcodes used for pre-indexing, and n represents the number of cells within a given droplet. To evaluate the effectiveness of usable cell recovery in each hashing strategy, we discarded the droplets containing more than one cell for the cellular hashing simulation. However, for molecular hashing, the droplets with a Tn5 barcode collision rate exceeding 10% were considered as undemultiplexable. As a result, the droplets containing more than 4 cells in a 96-pre-indexing system, those containing more than 3 cells in a 48-pre-indexing system, and those containing more than 2 cells in a 24-pre-indexing system were excluded from the count of usable cells.

**Comparison to dsciATAC**

The fastq files of dsciATAC datasets at 8,000, 16,000, 40,000, 80,000, and 160,000 nuclei inputs tagmented with 48 Tn5 barcodes were downloaded from the Gene Expression Omnibus (GEO) database under accession number GSE123581. The sequenced barcodes were parsed and assigned to the closest known sequence using a custom Python script allowing for up to one mismatch per 6-mer (Tn5) or 7-mer (Bio-Rad SureCell bead) barcode. The reads with corrected barcodes were then trimmed and mapped to a hybrid hg38/mm10 reference genome as described in the “Preprocessing'' section. To consolidate the bead barcodes within the same droplet for both dsciATAC and txci-ATAC data, we added the bead barcodes to a BAM tag, identified bead multiplets using the bap2 package, and then merged the inferred bead barcodes using a custom Python script that accounted for Tn5 barcodes. After this, the reads were filtered and deduplicated as detailed in the “Preprocessing” section, and subsequently used to quantify the species-specific mapping for each barcode ID (a combination of bead barcode, after merging bead barcodes from bead multiplets, and Tn5 barcode). To identify cell-associated barcodes within both dsciATAC and txci-ATAC datasets, we tested two distinct methods: 1) implementing a universal cutoff of 1,000 unique reads across all datasets, and 2) automatically determining a cutoff with *K*-means clustering for each dataset independently. For the latter approach, *K*-means clustering was applied to the log_10_-transformed unique reads from the barcode ID with a minimum of 100 unique reads using a value of *k* equal to two and 50 random sets. The collision rate was calculated as described in the “Collision rate estimation” section considering multiplets arising from either a single species or both species. For wells containing only cells from a single species in txci-ATAC-seq data, the multiplets were detected in each tissue separately using a customized Scrublet workflow, explained in the “Dimensionality reduction and clustering” section, and then eliminated prior to counting recovered cells. To conservatively identify singlets from those wells, if the multiplet rate inferred by Scrublet was lower than the empirical collision rate established from the wells designated as a true barnyard, the latter rate was used to remove the potential multiplets from the wells with pure species.”

**Reviewer reports:**

Reviewer #1: In their manuscript, "txci-ATAC-seq, a massive-scale single-cell technique to profile chromatin accessibility," Zhang, Mulqueen et al present a new single-cell ATAC-seq method that combines 10X barcoding (tx) and single-cell combinatorial indexing (sci) to increase the throughput of standard 10X scATAC experiments. The authors demonstrate that their approach works well on primary human and mouse tissue, and then perform a comparative experiment with wild-type and CC16-/- mice to establish gene regulatory differences in murine lung between these two models. The manuscript is well-written and the method seems robust, though the biological findings are surface-level. This manuscript is highly appropriate for the greater Genome Biology readership and I only have a few comments for the authors to address.

Comments
1)       The authors do not discuss why end-users might choose to carry out 'fast' txci-ATAC-seq versus standard txsci-ATAC-seq. Is the 'fast' version simply better in all respects? If so, this should be made explicit in the manuscript.

We appreciate the reviewer's comment. The rationale behind the development of the “fast” txci-ATAC-seq protocol stems from our objective to enhance the scalability of sample processing. To achieve this, we separated sampling processing from library preparation, enabling the transition from processing approximately a dozen samples (standard protocol) to efficiently handling up to 96 samples simultaneously within a single experiment (fast protocol). To make the name of this protocol variant more intuitive, we changed the name from Fast-txci-ATAC-seq to Phased-txci-ATAC-seq.

We have modified the following text in the manuscript on page 22 lines 420-431 to provide an explicit description of the advantages associated with the “phased” protocol:

“While the conventional txci-ATAC-seq method enables sample multiplexing, its efficiency is hindered by the labor-intensive nature of nuclei washing and counting procedures, constraining the number of samples (typically no more than 12) that can be processed in a single day. To address this limitation, we developed a “phased” protocol variant (Phased-txci-ATAC-seq) that effectively decouples sample processing from library preparation. Specifically, on the sample processing day, nuclei are isolated, quantified, and then cryopreserved in either PCR tube strips or a 96-well plate. On the designated library preparation day, the frozen nuclei are thawed and directly subjected to tagmentation via a modified transposition reaction. This alteration eliminates the need for intermediate steps such as nuclei washing and quantification on the library preparation day, empowering us to gradually accumulate samples (potentially up to 96) over time and subsequently process all samples in parallel during the library preparation phase of the protocol.”

2)       Please provide an estimate of the cost reduction provided by txsci-ATAC-seq compared to hashing, standard 10X ATAC-seq, or a commercial solution such as Scale Bio.

We thank the reviewer for the comment. We have added the following text to the Discussion section on page 29 lines 579-588 to compare the cost of txci-ATAC with other techniques:

“Based on current costs, we estimate that a standard 10X ATAC-seq run costs approximately $0.175 per cell. For txci-ATAC-seq, assuming that commercial Tn5 was purchased and loaded, the approximate cost is $0.015 per cell, representing a twelve-fold reduction in cost relative to the standard workflow. If one were to produce their own Tn5 following previously published protocols [54-56], the cost per cell would drop by 40% as we estimate that the commercial Tn5 accounts for $0.006 per cell of the total cost. We estimate that the cost per cell for cellular hashing would be approximately $0.047. In addition, cellular-level indexing results in a large number of cell multiplets, which cannot be used but still require extra costs for sequencing. Also, the antibody-based cell hashing approaches necessitate additional expenditures for acquiring hashing antibodies [49,50].”

3)       To maximize use of the datasets generated here, the authors should host and / or format their data for easy-browsing by readers using one of the available interactive data analysis frameworks for atlas browsing.

We appreciate the reviewer’s interest in enhancing the accessibility of our data. We completely agree that there are benefits to providing a web-based dataset for readers to easily browse through. Unfortunately, due to resource and personnel constraints, we are regrettably unable to commit to maintaining the required data and server for such an endeavor at this time. However, we are fully committed to upholding the transparency and accessibility of our research findings. In this regard, all of the data detailed in our paper will be made publicly available through GEO. Additionally, the code employed for analysis will be publicly accessible on GitHub and OSF, a DOI-assigning repository.

Reviewer #2: Genome Biology
txci-ATAC-seq, a massive-scale single-cell technique to profile chromatin accessibility
Hao Zhang et al.

The authors have modified an optimized a single-nuclei pre-indexing approach to be compatible with the 10X genomics platform downstream, for the purpose of achieving higher throughput compared to a standard 10X ATAC-seq workflow while preserving data quality. They tested their workflow, called txci-ATAC-seq, on several different sample types to demonstrate the validity of the method.

From the Genome Biology website description, "Method articles should describe novel methods that are shown to be a clear advance over existing state-of-the-art methods in a side-by-side demonstration, where possible." Overall, I do not deny the potential usefulness of this protocol for those researchers who wish to scale up snATAC-seq studies, but that only have access to the 10X platform (rather than the one previously reported by Lareau et al which uses the BioRad platform). However, I also feel that ultimately the innovation/novelty of this method, as well as the biological significance of the data reported herein, are limited. The general strategy of using pre-indexing has been reduced to practice previously by multiple groups, a point that the authors also discuss in their introduction section. Thus, the main novelty of this manuscript is to adapt this strategy to a different commercial platform. There is also some added value in the optimization of the SBS probes used to reduce Tn5 barcode swapping, but this is a technical point that I feel is not significant enough to justify publication in the current format. I am also uncertain that the data presented shows any clear advance over existing state-of-the-art methods. It is at best comparable to other published methods; but generally speaking, the level of benchmarking done in this manuscript is also insufficient, in terms of methods used for comparison and depth/thoroughness of analysis. The authors can refer to other benchmarking studies to see what types of analyses can and should be done to demonstrate performance. In terms of throughput, there are other methods that achieve much higher throughput.

We respectfully disagree with the notion that the novelty of our manuscript is limited and that our method is a simple optimization of an existing technique. To the best of our understanding, the only droplet-based method presently available for molecular hashing for single-cell ATAC-seq is dsciATAC-seq, a BioRad platform-based technique. There are numerous differences in the implementation of the two commercial platforms. For example, given the design of the barcoding beads, the Bio-Rad system requires an overloading of beads to reduce the number of empty droplets. This excessive bead loading, however, necessitates the consolidation of bead multiplets using computational inference and thus may introduce technical artifacts. By contrast, the 10X platform takes advantage of deformable hydrogel beads that can be closely packed in the microfluidic channels, achieving ~80% loading of one bead per droplet. In more carefully comparing the quality of data produced by the two methods, we have observed that txci-ATAC-seq consistently produces higher quality data (more complex with lower collision rate) from more cells than was reported in the original dsciATAC publication. In the end, while both methods rely on the same concept (a hybrid combinatorial indexing and microfluidics workflow), they are quite distinct in their actual implementation to the point that the dsciATAC protocol couldn’t serve as a starting point for implementing txci-ATAC-seq (beyond the conceptual insight).

Regarding the SBS probe, its inclusion was required to make txci-ATAC-seq viable with an acceptable collision rate. Furthermore, its role in preventing Tn5 barcode-swapping is not only relevant to single-cell ATAC-seq, but also holds the potential for broader applications in other single-cell techniques leveraging the 10x ATAC kit, such as single-cell CUT&Tag (<https://www.ncbi.nlm.nih.gov/pmc/articles/PMC7611252/>), which could potentially be transformed into a super-loading framework by incorporating the SBS probe.

As noted in the response to the Editor above, we have added several figures that address this comparison better. Furthermore, we have modified the following text on page 4 lines 76-83:

“However, this technique was formulated using the BioRad system, which requires an overloading of beads to reduce the frequency of empty droplets due to the design of the beads [10,11]. This bead overloading strategy necessitates the consolidation of bead multiplets using computational inference and therefore may introduce technical artifacts. In contrast, 10X Genomics, another commercialized platform widely embraced for single-cell data generation, takes advantage of deformable hydrogel beads that can be closely packed in the microfluidic channels, which allows ~80% loading efficiency of a single bead per droplet without overloading beads [12].”

As for the biological tissues profiled, some of these tissue types have previously been characterized/atlased in other publications (e.g., Duong et al, Cell genomics). The dataset using the CC16-/- mouse is potentially interesting, but the biological insights demonstrated are not validated by any further experimentation, thereby detracting from the significance of the study.

It's true that the lung tissue has also been characterized in Duong et al. 2022 as mentioned by the reviewer. However, that study carried out scTHS-seq in postnatal mice aged from postnatal day 1 to day 28. In contrast, our experiment was conducted in adult mice aged from 8 weeks to 24 weeks. In addition, the scale (n = 227,416 mouse lung cells) and quality of our data have enabled the identification of some rare cell types that were not characterized in Duong et al. 2022, such as the pulmonary neuroendocrine cells and basal cells. Thanks to the prompting of the Reviewer, we have added a reference to this study in our manuscript to emphasize the quality of our data.

We have added the following text on page 23 lines 454-457 to stress the improved power of our txci-ATAC in cell type identification, distinguishing it from other atlas efforts:

“While the cellular heterogeneity in mouse lungs has been characterized by other atlas efforts [45], the enhanced scale and quality of our data enabled the identification of certain rare cell types lacking well-defined regulatory DNA signatures, such as pulmonary neuroendocrine cells (PNECs) and basal cells.”

Regarding the biological significance of the knockout model, while we were initially very interested in the pathways implicated in the comparison between knockouts and wildtypes, the technical artifact we uncovered is confounded with those differences. While we think awareness of this artifact is important for the field (the original paper describing this mouse line has been cited 129 times), we did not think further investigation of the biological insights was warranted. We hope the Reviewer understands our reticence in this case. Nonetheless, we tried to extensively investigate the potential impact of the genetic variation present and have described the widespread and cell type-dependent effect of that variation, which again is an important theme to stress in the literature.

In my view, this manuscript can and should be published somewhere as it could be a useful method for some researchers, but it is not demonstrated to be a substantial improvement over existing methods and therefore doesn't quite fit the scope of Genome Biology Methods in my opinion; it may be more suitable as a protocols publication in a different journal. Or, if the authors generate significantly more biologically interesting analyses, it could become suitable.

With our new extensive evaluations in relation to SNuBar and dsciATAC, we hope that we’ve been able to convince the Reviewer that our method is a substantial improvement over existing methods.

In addition to the above comments, I also have some specific points for the authors' consideration:

1. Page 8 line 161 and line 172 - Lung barnyard and fragment size change after PCR data not shown - Is this an accepted practice at Genome Biology? For data that is very relevant to the study being described, I would like for it to be included in the SI at least. If there is some reason to not include it, please justify. If it is not important to the study, then it should be removed. To me it seems both these two pieces of data are interesting and relevant, and should be included in the SI.

We thank the reviewer for the suggestions. With regard to the “data not shown” for the lung barnyard, we were simply trying to be explicit that an estimate was missing because it could not be calculated with the method we implemented. To account for the probability of homotypic doublets (two cells from the same species), we inferred collision rates using the equation described in Bloom JD, 2018 (<https://pubmed.ncbi.nlm.nih.gov/30202659/>). For the “pseudo-barnyard” setting of lung tissues (originally page 8 line 161, now page 7 line 155), the equation resulted in an NA in the SBS condition because almost all the barcode-swapping events were blocked while only a single collision cell was observed. Since we visualized the blocking efficiency for each condition using a bar plot, we did not provide a separate plot to show the single collision cell we observed in the lung pseudo-barnyard. We apologize for the confusion. We agree with the reviewer that using a format of “data not shown” is not ideal. Therefore, we swapped the bar plot (Fig. S2c) with scatter plots to highlight all collision cells observed in each condition.

Below is the updated panel and figure caption for Fig. S2c:

c) Scatter plots showing the number of reads mapped to either human or mouse genome for the true barnyard (True) and pseudo-barnyard (Pseudo) settings with a mixture of GM12878 and CH12 cell lines or human and mouse lung cells at each blocking condition. Only mouse lung cells were loaded to the wells assigned to the “lung pseudo-barnyard”. The percentage represents the estimated collision rate (including doublets originating from the same species). The x- and y-axes are capped at 5x10^4^ reads, and the cells with at least 5x10^4^ reads are denoted by triangles.

Regarding the fragment size changes after PCR (originally page 8 line 172, now page 8 line 167), we did not show the data in the original manuscript because we tested the effect of PCR cycles on fragment sizes in an irrelevant project. But, we agree that this data is interesting and should be provided as a supplementary figure, so we conducted an additional experiment using a mixture of GM12878 and CH12 cells and amplified portions of the emulsion with either 3 cycles or 8 cycles of PCR during GEM incubation and reproduced our previously observed results.

Below are the TapeStation results and figure caption we have added to Fig S2g to show the fragment size distributions at different GEM PCR cycles:

g) TapeStation gel image showing the fragment size distributions at either 3 or 8 GEM PCR cycles. The first lane on the left indicates the DNA ladder.

2. For barnyard experiments, it is stated in the Methods section that they might also include the {multiplet single-species} cases, and that the collision events may be under-estimated as they would not include the multiplets that contain two nuclei from the same species. First, it would be helpful to be clear about this somewhere in figures/figure captions/in the text description of the results, rather than only in the Methods. Second, I am confused why the authors don't also calculate the estimated collision rate based on their barcoding, as a comparison to the modeling-based result. In addition, for mixed species cases, the authors could check for potential mis-barcoding using the species mapping result cross-checked against their barcoding demux result.

We thank the reviewer for the suggestion. Actually, we referred to “multiplets from single-species” as “homotypic doublets” in the original manuscript and mentioned it in the result section on page 7 from lines 129-130 (currently page 6 lines 124-125) as shown below:

“Unexpectedly, regardless of barnyard type (true vs. pseudo), the initial experiment exhibited an extremely high collision rate (including estimated homotypic doublets [11])”

To make this all clearer to the reader, we have included additional explanations in both text and figure captions as follows:

On page 6 lines 123-124, we have added the following text:

“To account for the collisions originating from the same species, all the collision rates reported in this study were estimated using the equation described in [14].”

We also removed “(including estimated homotypic doublets [11])” from page 6 line 125 to avoid redundancy with the above edits.

In the caption of Figure S2c, we have added:

“The percentage represents the estimated collision rate (including doublets originating from the same species).”

In the caption of Figure S5f (original Fig. 4c), we have added:

“The collision rate reported in each dataset includes doublets derived from the same species.”

Regarding the reviewer’s second question about why we don't calculate the estimated collision rate based on the barcoding information, it appears there might be some confusion with regard to our method and cell hashing approaches. In the context of cell hashing, the multiplets represent the cells that were brought into the same droplet and can be identified by quantifying the purity of hashtag oligo (HTO) species in each droplet. Given that the identified multiples are unable to be demultiplexed, the entire droplet enriching more than one HTO would be discarded in this approach.

In contrast, our method allows for accurate demultiplexing of cells that were co-encapsulated within the same droplet using their respective Tn5 barcodes, which renders them viable for subsequent analysis. The multiplets observed in our data that we were referring to, arise from the cells that originate from the same well on a 96-well plate and the same 10x droplet. As a result, these multiplets cannot be distinguished based on their barcode information, as they share identical Tn5 barcode (recording the well) and bead barcode (recording the droplet). That is why we adopted a mixed-species experiment to determine the multiplets.

3. Fig S2g shows some comparisons with other methods - would be helpful to include reference citations to those methods in the labels or elsewhere, as the names of each method may not be immediately recognizable to the reader.

We thank the reviewer for this suggestion and agree it would be helpful to the readability of the manuscript. We have added the author name and year for each data set to the figures and also included the accession number for each dataset in the Method section. Below we show the updated panel.

4. Fig 2c - motif images on the right side are too small and hard to read. Suggest moving to SI, or enlarging the figure somehow…

We have taken the suggestion of the reviewer and have limited the motifs shown in Fig 2c to the top 5 per cluster instead. We agree that this improves interpretability for the reader and thank the reviewer for the suggestion. Below is the updated figure.

5. Fig 3c and 3d - numbers on top of the bars overlap with graph lines and make it hard to read… also noted that Lareau et al.'s paper names their method that uses transpositional barcording dsciATAC-seq, not dscATAC which refers only to the use of droplets without overloaded barcoding approach. It would be important to clarify whether the comparison here is done using their combinatorial approach (dsci) or the regular approach (dsc), since the more comparable one would be dsci and not dsc, given that both are using barcoding to achieve overloading. In addition, I think it is important to see a more nuanced comparison of the data to other methods. For example, it is quite standard to perform correlation analyses to show the accuracy/precision when benchmarking against other methods (similar to Fig S1D and S1E in the SNuBAR paper by Kaile Wang et al, or Fig 1C and 1D in the dsciATAC paper by Lareau et al.).

We have added bounding boxes around the numbers to improve the readability of the figures. We opted for dscATAC-seq (dsc) dataset described in Lareau et al. because it used the same tissue type for direct comparison with our method (mouse brain). However, we would like to note that Lareau et al. describes in their paper that “dsc” seems to outperform “dsci”, so we believe the comparison we have performed is the most direct (in terms of being the same tissue type) and fair (as this value likely represents an overestimate of the performance of dsci). Below are the updated panels.

Fig. 3 c) Boxplots of FRiP per technology using a unified peak set. Numbers over the boxplot reflect the fold-change of medians in comparison to txci-ATAC-seq. Stippled line is the median value for txci-ATAC. d) Boxplots of transcription start site (TSS) enrichment across technologies. Numbers over the boxplot reflect the fold-change of medians in comparison to txci-ATAC-seq. Stippled line is the median value for txci-ATAC.

We have added additional analyses in the form of an additional supplementary figure showing both a genomic coverage plot across a pan-excitatory neuron gene *Slc17a7* split by technology. This shows that methods are consistent in read-pileups across accessible sites (“peaks”) identified across datasets. We also calculated a gene activity score through the count of Tn5 insertion fragments across all genes and correlated the average scores across the different single-cell ATAC technologies. This revealed a strong correlation across all technologies. A new supplementary figure (Fig. S4) is shown below. Overall we believe that with the addition of this supplementary figure showing consistency across datasets sufficiently demonstrates that txci-ATAC faithfully recapitulates the well-studied biology of the mouse brain. Especially as we reprocessed and called shared peaks across 8 different scATAC-seq datasets on a consistent tissue for direct comparisons.

We have added the following text to the manuscript on page 12 lines 250-256 to explain the new results:

“We found that the genomic coverage of ATAC-seq fragments across a pan-excitatory neuronal marker *Slc17a7* showed a similar distribution across compared technologies and our own (Fig. S4a). We further validated our technology's ability to capture reproducible chromatin accessibility patterns within the mouse brain by calculating the average gene activity score per gene and correlating our dataset to existing technologies via Spearman correlation (Fig. S4b). Our results revealed that all compared techniques generated consistent signals across genes (Spearman's rho ≥ 0.86 and p-value ≤ 0.001 for all comparisons).”

The detailed methods for the above analysis have been added on page 49 lines 1112-1119 and the citations for the packages used in the analysis have been provided on page 46 line 1015:

“Signac was then used to generate a SeuratObject as described above and a genomic coverage plot on aggregated technology signals was generated through the Signac function “CoveragePlot” as described previously for a pan-excitatory neuronal marker *Slc17a7* [21]. The number of ATAC fragments mapping in annotated genes or 2 kb upstream (promoter regions) was calculated via the Signac function “GeneActivity”, normalized by read depth per cell, and aggregated across methods. Transcripts greater than 500 kb were filtered out. Average gene and promoter coverage was then correlated across methods in a pairwise manner via Spearman correlation with R base function “cor”. Data were plotted via corrplot R package.”

**Supplementary Figure 4. Consistency of scATAC-seq mouse brain datasets.** a) Aggregated chromatin accessibility at the *Slc17a7* gene body and extended region (50,000 bp upstream and downstream) across methods. The aggregated accessibility signal for each cluster was normalized by a scaling factor computed as the number of cells in the cluster multiplied by the mean sequencing depth for the cells in that technology. Peaks called on all technologies aggregated are shown above and gene locations are shown below. b) Spearman correlation rho values shown across technologies in a pairwise fashion. Average gene accessibility (see Methods) was correlated and plotted both as a colored dot plot (top triangle) and as the rho value (bottom triangle). The distribution of the correlation coefficient for each technique was visualized using a violin plot.

6. Fig 3e - the result of the data integration appears unsatisfactory. For one, some of the clusters show these tentacle/splatter like features, which are indicative of bad clustering or bad data quality. Since this UMAP is not labelled with cell-type labels, it is hard to say which cluster is which cell type. But given the result shown in Fig 3a, and also the typical biology of the mouse brain, it is surprising that there are so many interconnected clusters (e.g. top left of the UMAP) in Fig 3e when this is not the case in Fig 1a. Furthermore, most clusters show poor integration of different datasets with data points being undermixed, and it is hard to tell if this is a data quality/fragments capture issue due to the methodology, or if it is an integration issue. In our own experience with RNA-seq data, we often experienced poor integration using Harmony on datasets generated by different methods/research groups); the performance is generally better for datasets with less batch effects such as biological replicates using the same method. While I understand that the purpose of this particular integration exercise is not necessarily to integrate these datasets perfectly, it should show that the authors' methods generate data that is comparable to other published methods/datasets. With such poor integration and the cell type labels missing, it is hard to judge whether this is indeed the case. Again, is the poor mixing of the integrated result due to systemic biases in the data itself, or an artefact of the integration algorithm? If the former, then some discussion of where the bias may arises would be important. If the latter, then it should be addressed before publication. There are many integration algorithms published, specifically for ATAC-seq data (note that Harmony is not - it is a general algorithm that CAN be used for ATAC but is not specifically designed for this data type). The authors may get better results with a more ATAC-specific tool.

We thank the reviewer for this comment and agree that better methods have been recently documented for scATAC-seq integration. Following the reviewer's suggestion, we found a recent meta-analysis paper (Leuken et al. 2022, <https://www.nature.com/articles/s41592-021-01336-8>) which performed comparisons across integration methods. We used their highest ranking method (LIGER on unscaled, binarized peaks, <https://www.cell.com/cell/fulltext/S0092-8674%2819%2930504-5> ) to redo the integration and saw a vast improvement.

We have updated the methods on page 50 lines 1120-1130 and figure panel (Fig. 3e) to show both the technology integration, and the cell type labels overlaid on the new integration. The citations for the packages used in the analysis have been added on page 46 line 1015. Below is the updated method:

“The integration across technologies was performed using LIGER, which was chosen for its performance as a top-performing method from a recent integration comparative study [99]. Briefly, the counts matrix was binarized and filtered to peaks with at least 50 cells showing accessibility, and cells were filtered to those with 500 or more peaks accessible. 3,695, 4,781, 3,031, 6,492, 906, 4,492, 8,295, and 38,605 scATAC profiles passed filters for sciATAC-seq, sciMAP, snATAC-seq, dscATAC-seq, tenxv1, tenxv2, s3-ATAC-seq, and txci-ATAC-seq, respectively. The top 50,000 peaks were chosen based on the “vst” method with the Signac function “FindVariableFeatures”. The remaining peaks and cells were then integrated with LIGER functions “RunOptimizeALS” with k=20, lamba=5, and split by method. LIGER function “RunQuantileNorm” was run and then the Signac function “RunUMAP” was used. The resulting integration was then plotted with Seurat “DimPlot” function for both cell type and method (Fig. 3e).”

Fig. 3 e) LIGER integrated UMAP projection of technologies (n = 75,845 cells) colored by technology (left) and cell type (right).

7. Figs 4 and 5 - the analysis here is missing some of the standard analyses typically used to evaluate/gain insight from ATAC-seq data, such as the marker gene accessibility landscape, signal enrichment around TSS, and motif analysis (which was done for the brain sample in Fig 2, but not done for the liver and lung).

We thank the reviewer for the suggestions. After carefully examining marker genes in both mouse lung and liver samples, we have updated some cell labels for the lung samples. For native lung samples in Fig. 4e (original Fig. 4g), we changed EPC (epithelial progenitor cell) to Basal1, Basal to Basal2, and intFB (interstitial fibroblasts) to vSMC (Vascular smooth muscle cells). For WT and CC16 KO lung samples in Fig. 5a, Fig. S13i (original Fig. S11f), and Fig. S14a (original Fig. S12a), we changed EPC to Basal and Epi-like (epithelial-like cells) to PNEC (Pulmonary neuroendocrine cells).

In response to the reviewer's request for the signal enrichment around the TSS, we want to clarify that we have indeed computed the per-cell TSS enrichment scores for both native (current Fig. 4c, the original figure number is Fig. 4e) and KO samples (current Fig. S13d, the original figure number is Fig. S11d) within the original manuscript. Despite this, at the reviewer's prompting, we have also added histograms to provide a visual representation of read density around the TSS for both liver and lung samples (detailed below).

Regarding the motif analysis, we have now identified the top accessible motifs in each cell type for both liver and lung samples (also detailed below).

Below are the detailed updates we have incorporated into the manuscript to show the marker gene accessibility landscape, signal enrichment around TSS, and motif analysis for liver and lung samples.

1) In Fig. S5g, we have added histograms to visualize the TSS enrichment for native mouse lung and liver samples and compare them with the TSS enrichment identified in sci-ATAC-seq data.

On page 16 line 298, we have added:

“the aggregated TSS enrichment increased from 10.7 to 22.2 for lung and from 7 to 18.2 for liver (Fig. S5g);”

We changed the title of Fig. S5 (original Fig. S4) to “Quality metrics of txci-ATAC-seq in the native lung and liver samples with loading 100,000 or 200,000 nuclei.”

The updated Fig. S5g and figure caption are shown as follows:

Fig. S5g. Distribution of scaled read depth within a 4000 bp window centered on genome-wide TSSs (with 2000 bp upstream and 2000 bp downstream) by aggregating all cells from lung (upper panels) or liver (lower panels) samples for each technique. The txci-ATAC data (red) combined the libraries of 100,000 and 200,000 loading inputs. X-axis indicates the base position relative to the TSS. Y-axis indicates the read depth for that position summarized across all annotated TSSs. The values are scaled relative to the mean coverage for the 100 bp at either end of this 4000 bp window.

2) We have added the dot heatmaps to highlight the marker genes and top accessible motifs in each cell type identified in native mouse lung (updated Fig. S6e,f) and liver samples (updated Fig. S7e,f).

From page 16 line 312 to page 17 line 323, we modified the main text to state:

“The associated cell type for each cluster was predicted by label transfer using previously published single-cell/single-nucleus RNA-seq (scRNA-seq/snRNA-seq) and sci-ATAC-seq datasets from mouse lung tissue (Fig. S6a-d; [3,30]), mouse liver tissue (Fig. S7a-d; [3,31]) and human lung tissue (Fig. S8; [32–34]). The predicted labels were further manually curated according to the gene activity scores (by summing the read counts in gene bodies and promoters [19]) of marker genes that exhibited cell-type-specific expression patterns using scRNA-seq data browsers of mouse lung (Fig. S6e; [[30,35]](https://paperpile.com/c/lsPqWS/Ycudc+fp7F)) and liver (Fig. S7e; [[31]](https://paperpile.com/c/lsPqWS/rApIT)). The top motifs in each cell type were also evaluated to further confirm the annotated cell types in the murine lung (Fig. S6f) and liver tissue (Fig. S7f). For example, the TEA domain (TEAD) motifs were highly accessible in AT1 cells [36]; tumor protein p63 (TP63) motif displayed increased accessibility in basal cells [37]; and hepatocyte nuclear factor (HNF) motifs were specifically accessible in hepatic lineage cells [38,39].”

We have added the following text to the Method section on page 60 lines 1373-1376:

“To identify differentially active motifs between cell types, a per-cell motif activity score was computed first for each motif using chromVAR implemented in Signac. The differential testing was then performed on the chromVAR z-score for each cell type using Seurat “FindAllMarkers” function.”

Below is the updated Fig. S6e and f for native lung:

Fig. S6 e,f. Dot plots of gene activity (e) and motif activity (f) scores for marker genes and top motifs identified using differential activity tests. One or two maker genes for each cell type were selected for plotting based on the specificity of their gene activity scores and restricted expression patterns observed using online scRNA-seq data browsers [[30,35]](https://paperpile.com/c/lsPqWS/Ycudc+fp7F). The motifs were chosen for plotting among those that were identified as significantly differentially accessible. The dot color depicts the scaled average gene or motif activity scores across all cells within a cell type, while the size of the dot encodes the percentage of cells in that cell type.

Below is the updated Fig. S7e and f for the native liver:

Fig. S7 e,f. Dot plots of gene activity (e) and motif activity (f) scores for marker genes and top motifs identified using differential activity tests. Two maker genes for each cell type were selected for plotting based on the specificity of their gene activity scores and restricted expression patterns observed using an online scRNA-seq data browser [31]. Two motifs were chosen for plotting among the significantly differentially accessible motifs identified for each cell type. The dot color depicts the scaled average gene or motif activity scores across all cells within a cell type, while the size of the dot encodes the percentage of cells in that cell type.

3) For CC16 deficient lungs, we have modified the following main text and added Fig. S13f-h (original Fig. S11) to show TSS enrichment and chromatin activity for marker genes and top motifs. According to the top marker genes, we have updated cell labels from EPC to Basal and from Epi-like to PNEC in Fig. 5a, Fig. S13i (original Fig. S11f), and Fig. S14a (original Fig. S12a)

On page 23 line 450, we have changed the figure annotation from “Fig. S11c-e” to “Fig. S13c-f” to incorporate the read density around TSS.

On page 23 lines 450-454, we have stated that:

“Using the iterative clustering strategy and label transfer with a scRNA-seq reference followed by manual curation with scRNA-seq marker genes [30, 35] (Fig. S13g) and top motifs (Fig. S13h), we identified 23 distinct cell clusters in mouse lungs profiled by the standard txci-ATAC-seq (Fig. 5a) and then used them to further annotate the Phased-txci-ATAC-seq lungs.”

Fig. S13 f) Distribution of scaled read depth within a 4000 bp window centered on the TSSs (with 2000 bp upstream and 2000 bp downstream) by aggregating all cells from either WT (upper panels) or CC16^-/-^ (lower panels) lung samples for each assay. X-axis indicates the base position relative to the TSS. Y-axis indicates the read depth for that position summarized across all annotated TSSs. The values are scaled relative to the mean coverage for the 100 bp at either end of this 4000 bp window. g,h) Dot plots of gene activity (g) and motif activity (h) scores for marker genes and top motifs identified using differential activity tests. One or two maker genes for each cell type were selected for plotting based on the specificity of their gene activity scores and restricted expression patterns observed using online scRNA-seq data browsers [30,35]. The motifs were chosen for plotting among those that were identified as significantly differentially accessible. The dot color depicts the scaled average gene or motif activity scores across all cells within a cell type, while the size of the dot encodes the percentage of cells in that cell type.

8. Fig 5 - for comparison between wt and -/- data, the authors might consider utilizing published bulk data or scRNA-seq data to gain more insight into the gene regulatory landscape caused by -/-, and whether these changes at the chromatin level that they observe can explain the RNA-seq observations.

We thank the reviewer for the suggestion and would like to clarify that our study has revealed a significant technical artifact, i.e., the remnant genetic material from the embryonic stem cells that were used to generate the knockout model has resulted in substantial changes in chromatin accessibility (and therefore gene regulation). This has led us to be cautious in interpreting strong biological insights from this model. While we did observe changes pertinent to the well-known CC16 phenotype, disentangling these from the technical artifact requires non-trivial effort. Therefore, we have chosen not to pursue further biological insights from this model. Importantly, the technical artifact unveiled in our study extends beyond the scope of the CC16 knockout model. it may exist in other knockout models developed in the same era. As a result, our study aims to raise awareness about this technical artifact caused by the residual genetic material and underscores the critical need to evaluate knockout models using a genome-wide genotyping approach.
